# Genetic analysis reveals unprecedented diversity of a globally-important plant pathogenic genus

**DOI:** 10.1038/s41598-019-43165-y

**Published:** 2019-04-30

**Authors:** Andrea R. Garfinkel, Katie P. Coats, Don L. Sherry, Gary A. Chastagner

**Affiliations:** Washington State University Puyallup Research and Extension Center, 2606 W, Pioneer, Puyallup, WA 98371 USA

**Keywords:** Agroecology, Biodiversity, Microbial ecology

## Abstract

Genus *Botrytis* contains approximately 35 species, many of which are economically-important and globally-distributed plant pathogens which collectively infect over 1,400 plant species. Recent efforts to genetically characterize genus *Botrytis* have revealed new species on diverse host crops around the world. In this study, surveys and subsequent genetic analysis of the glyceraldehyde-3-phosate dehydrogenase (*G3PDH*), heat-shock protein 60 (*HSP60*), DNA-dependent RNA polymerase subunit II (*RPB2*), and necrosis and ethylene-inducing proteins 1 and 2 (*NEP1* and *NEP2*) genes indicated that *Botrytis* isolates collected from peony fields in the United States contained more species diversity than ever before reported on a single host, including up to 10 potentially novel species. Together, up to 16 different phylogenetic species were found in association with peonies in the Pacific Northwest, which is over a third of the total number of species that are currently named. Furthermore, species were found on peonies in Alaska that have been described on other host plants in different parts of the world, indicating a wider geographic and host distribution than previously thought. Lastly, some isolates found on peony share sequence similarity with unnamed species found living as endophytes in weedy hosts, suggesting that the isolates found on peony have flexible lifestyles as recently discovered in the genus. Selected pathogenicity, growth, and morphological characteristics of the putatively new *Botrytis* species were also assessed to provide a basis for future formal description of the isolates as new species.

## Introduction

Fungi in genus *Botrytis* are economically-important agricultural plant pathogens that collectively infect nearly 600 diverse plant genera comprising 170 monocotyledonous and dicotyledonous plant families^1^. Many of these species putatively infect only a restricted range of host plant species, however, the polyphagous *B. cinerea* alone has been documented to cause disease on 586 genera^1^. This species can also be found infecting plants in remote locations from Hawaii^2^ to the Canary Islands^3^, and has even been recorded from environmental samples in Antarctica^4^, making the influence of this pathogen truly global. *Botrytis* species are present in all major agricultural production regions of the world and cause significant pre- and post-harvest losses, supporting a multi-million dollar *Botrytis* pest management industry.

The importance of *Botrytis* extends beyond the agricultural field. *B. cinerea* is widely utilized as a chief model organism for understanding plant-pathogen interactions such as the evolution of necrotrophy^5^ and host-specificity^6^, and the flexibility of plant pathogen lifestyles^7^. Recent investigations into the ability of *B. cinerea* to exist as endophytes while maintaining virulence^7^, and the plant-pathogen transcriptome-level interactions involved in this fascinating exchange^8^, are effectually destroying traditional, rigid classifications of plant-associated fungi as either pathogens, endophytes, or saprophytes^7^. The ability of fungi to suppress the plant immune response by small RNAs, arguably one of the most monumental advances in the understanding of plant-microbe molecular interactions in the recent decade, was first discovered in *B. cinerea*^9^.

Since the initial description of the genus in 1729, over 35 species of generalist (polyphagous) and host-specific *Botrytis* species have been described^1,10–23^, with a recent upswing in the number of species described due to molecular advances. Twelve species of *Botrytis* have been formally described since 2010, for an average rate of more than one per year over the last 8 years^10,11,13–19,21–23^. New *Botrytis* species have been found worldwide from China^10,21–23^, to Germany^17^, to Chile^11^, and on wide range of host plants such as onion (*Allium cepa*)^22^, blueberries (*Vaccinum corymbosum*)^18^, and daylily (*Hemerocallis* hybrids)^14^. New species descriptions are supported by phylogenetic analysis of 5 genes most widely used and accepted for species delineation in *Botrytis*: glyceraldehyde-3-phosate dehydrogenase (*G3PDH*), heat-shock protein 60 (*HSP60*), DNA-dependent RNA polymerase subunit II (*RPB2*), and necrosis and ethylene-inducing proteins 1 and 2 (*NEP1* and *NEP2*)^6,20,24,25^. Species delineation by morphological characterization alone is difficult in *Botrytis* due to overlap in the size of fungal structures among species, morphological plasticity within species and individual isolates, and variable phenotypic expression under diverse cultural conditions^20^. The surge in species recognition across regions and hosts clearly indicates that undiscovered and cryptic *Botrytis* species, those that are indistinguishable from each other based on morphology, exist at high frequencies, placing *Botrytis* taxonomy at what has been described as an “early stage”^20^.

We conducted surveys in the states Washington, Oregon, and Alaska from 2014–2015 in an effort to genetically characterize the species of *Botrytis* present on the specialty crop *Paeonia lactiflora* (peony) in the Pacific Northwest of the United States. Previously, Botrytis grey mould of peonies has been reported to be caused by four species of *Botrytis*: *B*. *cinerea*, described above; *B. paeoniae*, a putatively host-specific pathogen of peonies present in most areas where peonies are grown^1,26–28^; *B. pseudocinerea*, a recently described so-called “cryptic” species of *Botrytis* that is only distinguishable from *B. cinerea* through genetic analysis and fenhexamid fungicide sensitivity^19,27,29^; and *B. euroamericana*, a fungus found on both peony and grape that was reported from Alaska and Italy, respectively^13^. We discovered additional isolates that appear to be novel *Botrytis* species on peonies that collectively represent up to a 30% increase in the known genetic diversity of this genus, including a number of isolates that also appear to be genetically similar to species that have been recently described and those for which only sequence data are currently available and no official taxonomic description has been produced. We report herein phylogenetic diversity in genus *Botrytis* that is yet unprecedented in other surveys that have been conducted throughout the world on other host crop species.

## Results

**Figure 1:**
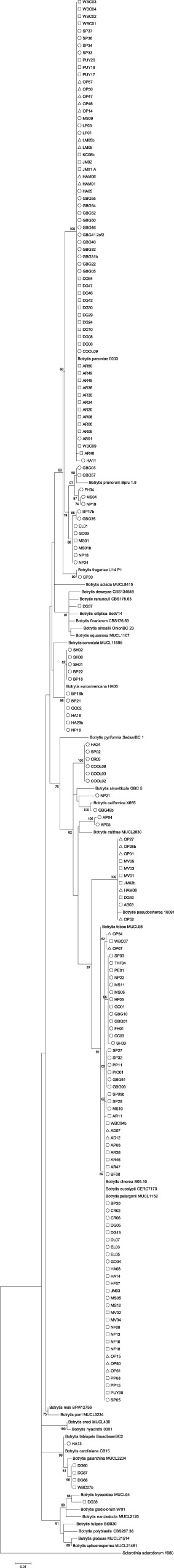
Maximum likelihood tree of *G3PDH* gene sequences. The tree describes the relationship of *Botrytis* species isolates collected from peonies in Alaska (indicated by ○), Oregon (indicated by Δ), and Washington (indicated by □) to named *Botrytis* species using *Sclerotinia sclerotiorum* as an outgroup. A total of 875 positions were used in the final dataset. Evolutionary relationships were modelled using a Kimura 2-parameter model with gamma distribution rates. Bootstrap percentages (n = 1000) are shown on branches. Branches with <50% bootstrap support are not shown. The tree is drawn to scale with branch lengths proportional to the number of substitutions per site.

A total of 178 *Botrytis* isolates were collected from peonies in three states in the Pacific Northwest of the United States. The *G3PDH* gene was sequenced for all 178 isolates from peony and an individual gene tree was constructed (Fig. 1). Of the 178 *Botrytis* isolates collected from peony, 136 isolates (76.4%) were identified as either *B. cinerea*, *B. paeoniae*, or *B. pseudocinerea* according to their co-occurrence in a well-supported clade with voucher sequences for these species in the individual *G3PDH* gene tree (Fig. 1). Within the subset of isolates that were one of these three species, 62 were identified as *B. cinerea* (34.8% of the total isolates collected), 63 as *B. paeoniae* (35.4% of the total isolates collected), and 11 as *B. pseudocinerea* (6.2% of the total isolates collected) (Fig. 1). The remaining 42 *Botrytis* isolates (23.6%) did not genetically align with any of these three species. Of the 42 isolates that do not appear to be one of these three species, 36 are from Alaska and 6 are from Washington; none of the isolates from Oregon did not correspond with one of these three species (Fig. 1).

**Figure 2:**
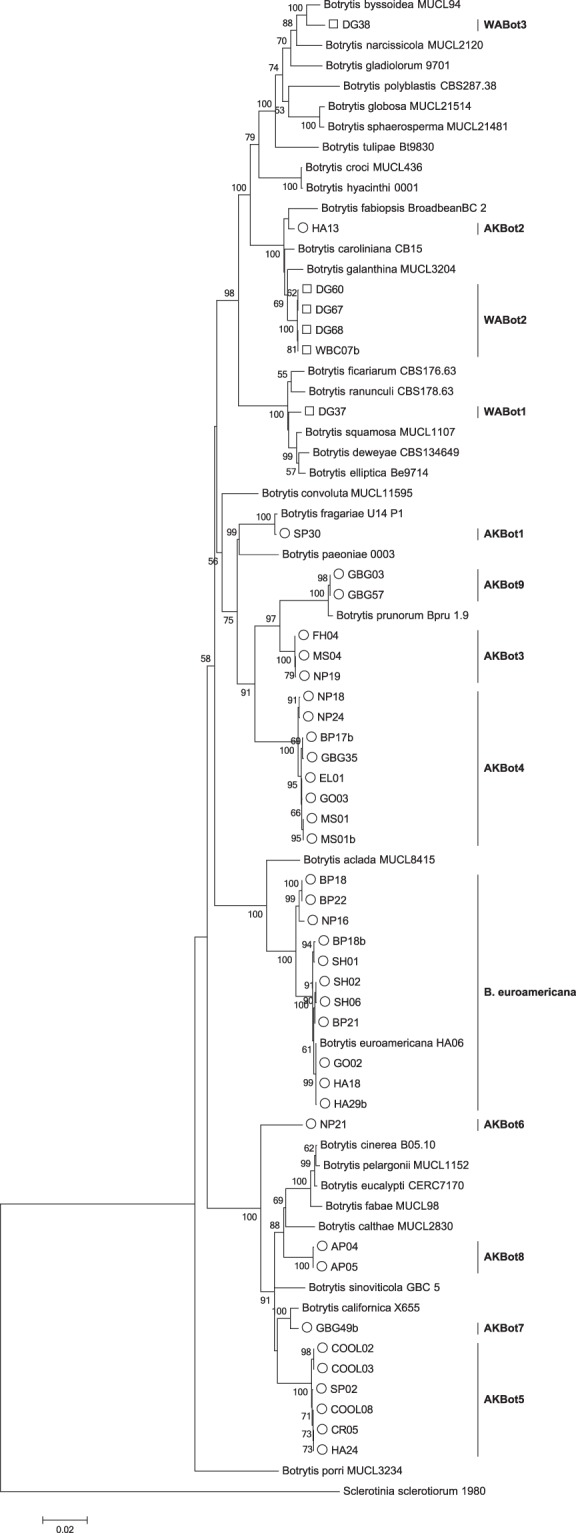
Maximum likelihood tree of combined *G3PDH*, *HSP60*, *RPB2, NEP1*, and *NEP2* gene sequences. The tree describes the relationship of *Botrytis* species isolates collected from peonies in Alaska (indicated by ○) and Washington (indicated by □) to named *Botrytis* species using *Sclerotinia sclerotiorum* as an outgroup. A total of 4,336 positions were used in the final dataset. Evolutionary relationships were modelled using a General Time Reversible model with gamma distribution rates and invariant sites. Bootstrap percentages (n = 1000) are shown on branches. Branches with <50% bootstrap support are not shown. The tree is drawn to scale with branch lengths proportional to the number of substitutions per site.

The subset of isolates that did not cluster into phylogenetic clades with *B. cinerea*, *B. paeoniae*, or *B. pseudocinerea* based on results using only the *G3PDH* gene were subject to additional sequencing and a phylogenetic analysis of combined data sets of the *G3PDH* + *HSP60* + *RPB2* + *NEP1* + *NEP2* genes (Fig. 2). Individual gene trees used to construct the concatenated data sets demonstrated similar branching patterns, with the exception of a difference in the *NEP1* tree for isolates BP18, BP22, and NP16. Nevertheless, the similarities among all other isolates provided sufficient justification for concatenation. The individual gene trees are available for comparison as electronic Supplementary Information (Supplementary Figs S1–S5). Combined *G3PDH* + *HSP60* + *RPB2* + *NEP1* + *NEP2* gene trees indicated that 8 of the 42 isolates, all from Alaska, grouped into a well-supported clade with *B. euroamericana*, a species recently described from peony in Alaska^13^ (Fig. 2). Although isolates BP18, BP22, and NP16 separate from *B. euroamericana* in the 5-gene phylogeny, these isolates may also be representatives of this species. The separation is likely a result of the differences in the aforementioned differences in the *NEP1* gene (Supplementary Fig. S4); intraspecific variation has been shown to exist in the *NEP1* gene of other *Botrytis* species^24^. Two isolates from Alaska (GBG03 and GBG57) grouped into a clade with *B. prunorum*, a species described on plum (*Prunus*) in Chile^11^ (Fig. 2, Supplementary Figs S1–S5). Isolate SP30 from Alaska appeared to be the same as or closely related to *B. fragariae*, a species described on strawberries in Germany and in the East Coast of the United States^17,30^. The remaining isolates (n = 28) did not appear to phylogenetically align with other named species and appear to represent novel clades within genus *Botrytis* (Fig. 2, Supplementary Figs S1–S5). In Fig. 2, the various phylogenetic clades represented by our samples have been designated as AKBot1-9 for the samples from Alaska and WABot1-3 for the samples from Washington and are referred to as such throughout the results section.

**Figure 3:**
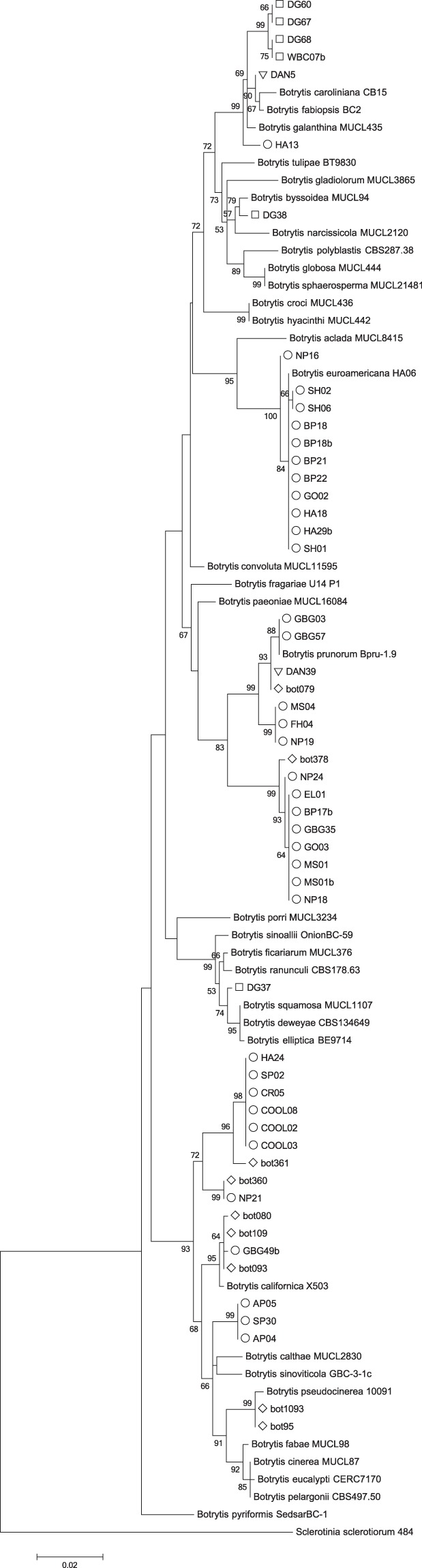
Maximum likelihood tree of *HSP60* gene sequences. The tree describes the relationship of *Botrytis* species isolates collected from peonies in Alaska (indicated by ○) and Washington (indicated by □) to named *Botrytis* species and endophytic *Botrytis* species from *Centaurea stoebe* (indicated by ◊) (Shipunov *et al*.^31^) and *Taxacum officinale* (indicated by ▿) (Shaw *et al*.^32^) using *Sclerotinia sclerotiorum* as an outgroup. A total of 834 positions were used in the final dataset. Evolutionary relationships were modelled using Kimura 2-parameter model with gamma distribution rates. Bootstrap percentages (n = 1000) are shown on branches. Branches with <50% bootstrap support are not shown. The tree is drawn to scale with branch lengths proportional to the number of substitutions per site.

The *HSP60* gene sequences of peony *Botrytis* isolates were also compared to unnamed endophytic *Botrytis* species collected from the invasive plant species *Centaurea stoebe*^31^ and *Taraxacum officinale*^32^. The analysis showed that two peony isolates from Alaska grouped into clades with isolates from *C*. *stoebe* (Fig. 3). Alaska peony *Botrytis* isolate NP21 was genetically similar to *C. stoebe* isolate bot360 and peony *Botrytis* isolate GBG49b appeared to be genetically similar *C. stoebe* isolates bot080, bot109, and bo093 (Fig. 3). According to this phylogenetic analysis, two additional *C. stoebe* isolates (bot1093 and bot095) aligned with *B. pseudocinerea* (Fig. 3), a species described after the survey of *C. stoebe* endophytes, while the remaining three *C. stoebe* isolates (bot 079, bot378, and bot361) did not appear to be genetically similar to any described species or isolates collected in this study. None of the *Botrytis* isolates from peony grouped into clades with isolates DAN5 or DAN39 (putatively identified by the authors as *B. mali*) from *T. officinale*^32^ (Fig. 3). DAN39 did however group with *C. stoebe* isolate bot079, as previously reported^32^.

Finally, an additional phylogenetic analysis of combined *G3PDH* + *HSP60* gene sequences was performed on 11 *Botrytis* isolates from peony that were identified as *B. pseudocinerea* to determine to which genetic clade the isolates belong. Isolates of *B. pseudocinerea* have been shown to be genetically diverse and fall into two major groups^29,33^. The isolates from peony were compared to 13 reference *B. pseudocinerea* isolates comprising both gnetic groups and relevant outgroups (Supplementary Table S2). The phylogenetic analysis showed that all the *B. pseudocinerea* isolates from peony grouped with reference *B. pseudocinerea* isolates previously described as *B. pseudocinerea* group A^29^ (Supplementary Fig. S6). *Botrytis* isolates AP04 and AP05 from peony in Alaska were also included in the phylogenetic analysis given their close relatedness to *B. pseudocinerea* with the purpose of confirming whether or not these isolates are representatives of this species. The additional analysis confirms that isolates AP04 and AP05 group into a novel clade more closely related to *B. cinerea* and *B. calthae* than to *B. pseudocinerea* and are therefore not likely representatives of this species (Supplementary Fig. S6).

**Figure 4:**
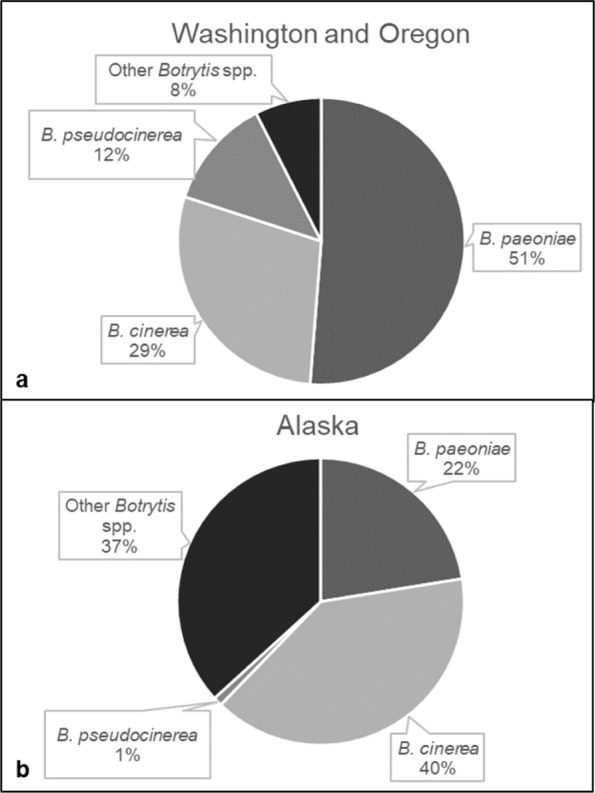
Breakdown of isolates of *Botrytis* species collected from peony. Isolates were collected from (**a**) Washington and Oregon (n = 80) and (**b**) Alaska (n = 98).

The relative frequency of *Botrytis* species collected from peony differed by survey region (Fig. 4). In Washington and Oregon (combined), the majority of isolates were identified as *B. paeoniae* (51%), followed by *B. cinerea* (29%) (Fig. 4). Only 8% of the isolates (n = 6) collected in Washington and Oregon (all of which were collected in Washington) did not belong to either *B. cinerea*, *B. paeoniae*, or *B. pseudocinerea* (Fig. 4). On the contrary, 37% of the isolates found in Alaska were species other than *B. cinerea*, *B. paeoniae*, or *B. pseudocinerea*, closely followed by isolates that were identified as *B. cinerea* (40%) (Fig. 4).

**Figure 5:**
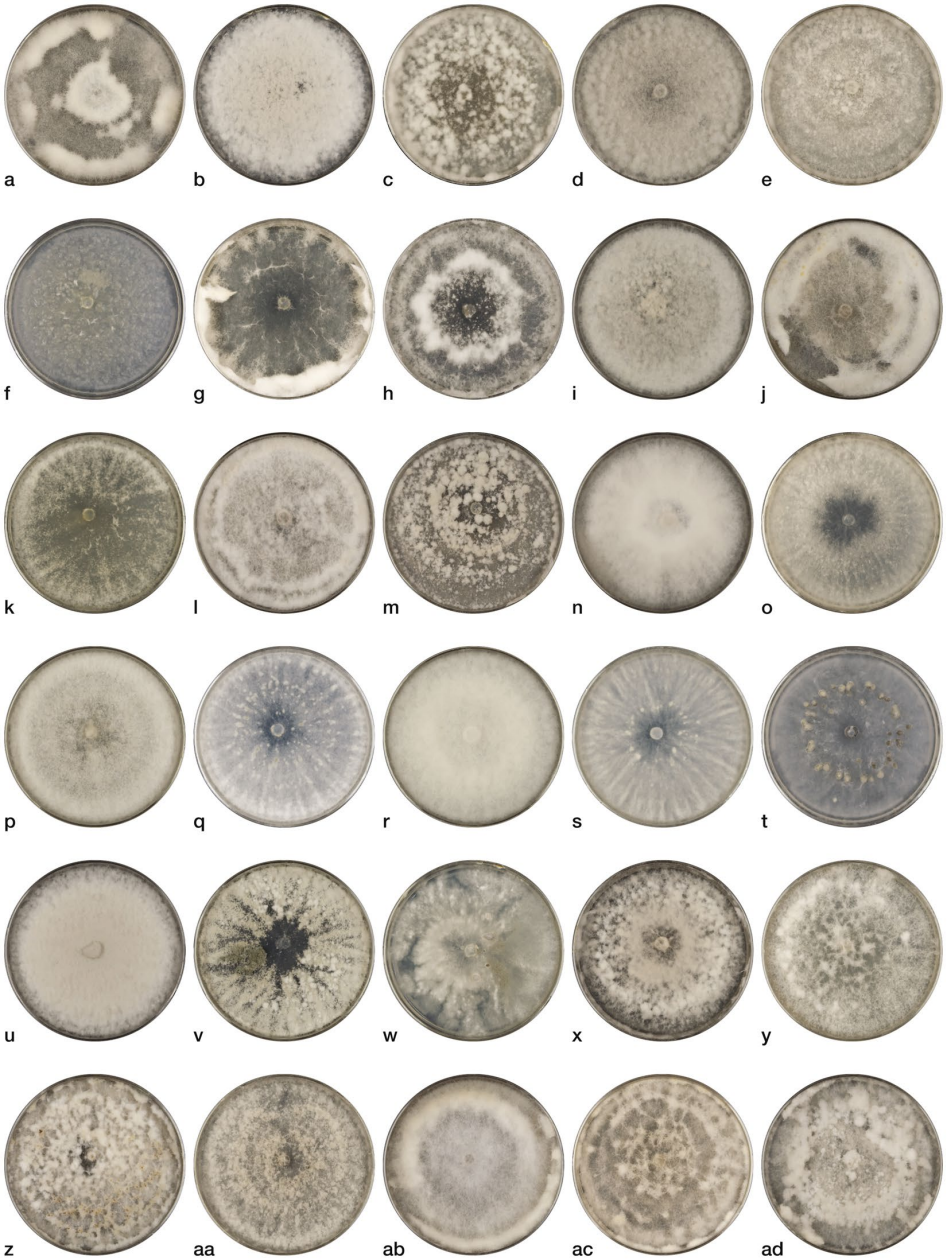
*Botrytis* spp. isolates (**a**) SP30, (**b**) HA13, (**c**) FH02, (**d**) MS04, (**e**) NP19, (**f**) BP17b, (**g**) EL01, (**h**) GBG35, (**i**) GO03, (**j**) MS01, (**k**), MS01b, (**l**) NP18, (**m**) NP24, (**n**), COOL02, (**o**) COOL03, (**p**) COOL08, (**q**) CR05, (**r**) HA24, (**s**) SP02, (**t**) NP21, (**u**) GBG49b, (**v**) AP04, (**w**) AP05, (**x**) GBG02, (**y**) GBG57, (**z**) DG37, (aa) DG60, (ab) DG67, (ac) DG68, and (ad) WBC07c collected from peonies grown on potato dextrose agar for 7 days in the dark at 20 C.

In order to obtain preliminary morphological characterizations of the putatively new species, isolates were grown up on potato dextrose agar (PDA) and malt extract agar (MEA) in the dark and under constant near ultra-violet (UV) light (350 nm) and on *Botrytis*-specific media (BSM)^34^ in the dark, all at 20 C. Colonies were diverse in colour and form, both among and within phylogenetic clades, and differed slightly depending on growth media. Colours on PDA and MEA ranged from pure white, to off-white, to tan or grey. Hyphae grew flat and appressed to the agar surface in some isolates and floccose, tufted, and/or woolly in others (Fig. 5, Supplementary Figs S7, S8). Notable colony morphology characteristics included: a radiating, stellate growth pattern of isolates MS04, NP19, and FH04 evident on MEA in the dark (Supplementary Fig. S9), but not on MEA under UV light or PDA in either light regime (Fig. 5, Supplementary Figs S7, S8); formation of melanised sclerotia in isolates of DG60, NP21, and AP05 at 7 days grown on PDA in the dark (Fig. 5), earlier than any other isolates tested, but not evident in isolates grown on MEA or under constant UV light; and a powdery growth pattern of isolate AP04, evident in all cultures grown under all media-light combinations. Isolates AP04 and AP05 also showed evidence of greater sporulation in all media-light combinations than any other putatively new species isolates tested (Fig. 5, Supplementary Figs S7, S8). Sporulation was rare to absent in most isolates on both PDA and MEA at 4, 7, 14, and 21 days in both the dark and under UV light. All isolates had less sporulation as compared with *B. cinerea* isolate MS05^13^ grown as a comparison, which sporulated most profusely under UV light conditions. Isolate DG38 failed to grow after being transferred for growth characterization trials, therefore this isolate is not represented in any of the growth or pathogenicity trials conducted for this paper. Later attempts to revive this isolate from the original archived culture were successful and therefore a photograph of this isolate is presented as Supplementary Fig. S10.

All isolates tested were able to grow hyphae across a *Botrytis*-specific medium^34^ (BSM) used in other studies for the isolation and characterization of *Botrytis* species^32,35–37^. Although all of the isolates tested were able to grow, there were marked differences in the colony morphology and extension across the agar surface. Some isolates produced dense, cottony growth, while others produced scant individual hyphae, sometimes forming into tufts (Supplementary Fig. S11). All isolates stained the pink agar surface to brown in a radial pattern that extended slightly past observable hyphal growth (Supplementary Fig. S11). *B. cinerea* isolate MS05^13^ was grown on BSM as a comparison. This isolate produced prolific conidia on BSM that landed and produced new colonies on the agar surface. This pattern was not seen in any of the putative new *Botrytis* species tested. Isolates CR05 and NP21 produced sclerotia on BSM after 14 and 21 days, respectively; no other isolates produced sclerotia in the time these cultures were observed.

Growth of selected isolates were also assessed at 7 temperatures in 5-degree increments ranging from 5 to 35 C. Temperature trials indicated that all isolates tested grew fastest at either 20 or 25 C (Table 1). Growth was significantly reduced at 5, 10, and 30 C for all isolates, with no growth occurring at 30 C for isolates AP04 and AP05. No growth was observed for any of the isolates at 35 C. Notably, growth resumed in several isolates that had been incubated at 35 C once transferred back to 20 C for 4 to 5 days (Table 1). However, growth was not resumed in isolates HA24, COOL08, COOL03, or COOL02, and only one replicate (n = 6) of SP02 and CR05 resumed growth after being transferred to lower temperatures (Table 1). All of these isolates are representatives of phylogenetic group AKBot8. Growth was also not resumed upon transfer to 20 C following incubation at 35 C for a number of additional isolates from both Alaska and Washington (Table 1).

**Table 1:** Average colony diameter (mm) of *Botrytis* species collected from peony after incubation at different temperatures for 48 hours.

Species	Isolate	Temperature (C)							Recovery
		5	10	15	20	25	30	35	
AKBot1	SP30	15.6c	18.3c	28.8b	35.2a	31.5ab	10.1d	0e	0
AKBot2	HA13	20.2d	25.1c	36.8b	43.1a	36.3b	14.3e	0f	0
AKBot3	FH04	12.2 f	21.5e	36.6c	51.6b	54.8a	26.6d	0g	6
	MS04	12.0d	20.6c	35.1b	51.5a	53.3a	20.2c	0e	6
	NP19	12.3e	20.6d	36.3b	53.2a	53.8a	22.9c	0f	6
AKBot4	BP17b	10.9 f	19.3e	30.3c	41.4b	46.2a	27.3d	0g	6
	EL01	14.4e	24.2c	35.6b	44.9a	45.8a	21.8d	0f	6
	GBG35	11.6 f	20.9e	32.3c	46.2b	51.8a	23.3d	0g	6
	GO03	13.7d	24.0c	36.3b	48.3a	50.8a	22.7c	0e	6
	MS01	12.6d	21.2c	32.0b	39.6a	36.2ab	21.4c	0e	5
	MS01b	10.4d	19.6c	25.8b	33.1a	35.3a	26.0b	0e	6
	NP18	8.1e	16.6d	25.2c	33.9b	37.3a	15.6d	0f	6
	NP24	10.3 f	21.5e	34.8c	50.0b	54.3a	27.7d	0g	6
AKBot5	COOL02	16.4d	25.0c	38.4b	46.7a	48.0a	11.4e	0f	0
	COOL03	16.3d	26.6c	39.0b	49.5a	48.8a	12.3e	0f	0
	COOL08	11.9d	21.8c	33.6b	42.7a	39.0a	8.6d	0f	0
	CR05	16.1e	27.4d	36.6c	47.4b	52.1a	11.8 f	0g	1
	HA24	15.7d	25.9c	39.8b	51.5a	52.1a	13.0e	0f	0
	SP02	14.8d	26.6c	38.7b	49.3a	49.3a	12.3d	0e	1
AKBot6	NP21	20.1e	29.0c	40.8b	52.1a	50.7a	23.8d	0f	0
AKBot7	GBG49b	19.5e	25.8d	39.1c	49.5a	44.1b	14.3 f	0g	0
AKBot8	AP04	19.6e	27.3d	41.5b	52.8a	34.4c	0 f	0f	0
	AP05	16.6c	28.6b	35.6b	45.8a	28.8b	0d	0d	0
AKBot9	GBG03	9.7e	18.5d	32.6c	46.9b	51.2a	16.9d	0f	6
	GBG57	11.5 f	19.1e	31.3c	42.8b	46.3a	24.8d	0g	6
WABot1	DG37	15.3d	21.7c	31.7b	39.7a	37.1a	11.7e	0f	0
WABot2	DG60	12.8e	19.6d	33.6c	46.3b	48.8a	17.5d	0f	6
	DG67	11.2e	18.4d	30.3b	38.9a	39.1a	20.7c	0f	6
	DG68	9.7d	18.3c	30.5b	46.0a	49.2a	18.8c	0e	6
	WBC07b	11.7d	18.6c	30.8b	45.7a	42.9a	20.4c	0e	6

Values followed by the same letter in a row are not statistically different (*p* = 0.05). Recovery represents the number of plugs (n = 6) with growth after transfer from 35 C to 20 C. Isolates AP04 and AP05 did not grow at 30 C, but resumed growth upon transfer to 20 C.

Initial pathogenicity trials indicated a range in abilities of the isolates to cause disease on detached peony leaves. In some cases, lesion development was consistent and their sizes comparable to known peony pathogens *B. cinerea* and *B. paeoniae*, whereas in other instances, no lesions were evident in the preliminary trials conducted. All isolates in phylogenetic groups AKBot5 (COOL02, COOL03, COOL08, CR05, HA24, and SP02) and AKBot8 (AP04 and AP05) consistently caused expanded lesions on peony cultivars ‘Kansas’ and ‘Sarah Bernhardt’ (Table 2), some of the most popular cultivars grown in the US cut flower industry. Isolate NP21 (AKBot6), also showed a relatively consistent ability to cause expanding lesions on host tissue, but lesions were more frequently seen on ‘Kansas’ than on ‘Sarah Bernhardt’ (Table 2). Isolates that showed no ability to cause disease in any of the trials conducted on either cultivar included DG37 (WABot1) and WBC07b (WABot2). The remaining isolates caused lesion development greater than the diameter of the plug used for inoculation at least once in all of the trials conducted, however, with variability among trials repeated on the same cultivar and among cultivars (Table 2).

**Table 2:** Lesion development on ‘Kansas’ and ‘Sarah Bernhardt’ peony leaves inoculated with *Botrytis* isolates.

Species	Isolate code	Symptom	Kansas				Sarah B			
			Trial 1		Trial 2		Trial 1		Trial 2	
			Avg. lesion dia (mm)	No. of leaves with lesions (n = 3)	Avg. lesion dia (mm)	No. of leaves with lesions (n = 3)	Avg. lesion dia (mm)	No. of leaves with lesions (n = 3)	Avg. lesion dia (mm)	No. of leaves with lesions (n = 3)
AKBot1	SP03	Blasted bud	14.0	1	23.5	1	0.0	0	0.0	0
AKBot2	HA13	Flower bud decay	16.0	1	0.0	0	12.5	1	22.0	1
AKBot3	FH04	Leaf tip dieback	13.0	2	0.0	0	12.2	3	0.0	0
	MS04	Blasted bud	9.0	1	0.0	0	8.5	3	0.0	0
	NP19	Blasted bud	10.5	2	9.0	1	18.2	3	0.0	0
AKBot4	BP17b	Shoot dieback	13.7	3	12.0	1	12.5	1	9.0	1
	EL01	Foliar lesion	0.0	0	0.0	0	10.3	3	0.0	0
	GBG35	Foliar lesion	0.0	0	0.0	0	8.5	2	11.0	2
	GO03	Flower bud decay	13.0	1	0.0	0	8.3	3	0.0	0
	MS01	Flower bud decay	0.0	0	0.0	0	10.8	3	0.0	0
	MS01b	Shoot dieback	10.5	3	11.0	1	9.5	2	0.0	0
	NP18	Blasted bud	13.0	1	0.0	0	7.8	2	0.0	0
	NP24	Foliar lesion	0.0	0	9.5	1	7.5	1	0.0	0
AKBot5	COOL02	Foliar lesion	37.7	3	23.2	3	23.3	3	35.2	3
	COOL03	Foliar lesion	18.0	3	23.8	3	13.5	3	29.7	3
	COOL08	Foliar lesion	25.0	3	19.7	3	10.0	3	26.0	3
	CR05	Foliar lesion	20.8	3	21.8	3	24.0	3	30.3	3
	HA24	Shoot dieback	34.2	3	16.5	1	27.5	3	23.0	3
	SP02	Flower decay	29.2	3	22.0	3	15.5	3	27.5	3
AKBot6	NP21	Leaf tip dieback	‘22	2	12.5	2	17.5	1	32.5	1
AKBot7	GBG49b	Lesion at petiole	0.0	0	24.5	0	32.5	1	27.5	1
AKBot8	AP04	Blasted bud	26.2	3	38.0	3	0.0	0	29.2	3
	AP05	Foliar lesion	36.5	3	53.3	3	38.2	3	48.0	3
AkBot9	GBG03	Basal stem decay	12.0	2	9.5	1	14.0	1	0.0	0
	GBG57	Shoot dieback	12.8	3	0.0	0	16.5	1	0.0	0
WABot1	DG37	Foliar dieback	0.0	0	0.0	0	0.0	0	0.0	0
WABot2	DG60	Foliar dieback	0.0	0	0.0	0	0.0	0	8.5	1
	DG67	Blasted bud	18.0	1	0.0	0	0.0	0	0.0	0
	DG68	Blasted bud	0.0	0	0.0	0	8.5	3	0.0	0
	WBC07b	Leaf tip dieback	0.0	0	0.0	0	31.0	1	0.0	0
*B. cinerea*	MS05	Flower decay	14.7	3	23.7	3	30.0	3	21.5	3
*B. paeonia*	AR05	Basal stem decay	58.7	3	48.3	3	0.0	0	64.5	3

Plugs of potato dextrose agar colonized by the *Botrytis* species were placed onto detached peony leaves and incubated at 20 C in the dark. Due to differences in rate of leaf colonization among trials, data are from 4, 6, or 7 days post inoculation. Symptoms describe the original host tissues from which the isolates were obtained.

## Discussion

Surveys of peonies in Alaska, Washington, and Oregon revealed isolates that did not belong to any of the previously reported *Botrytis* species and can preliminarily be classified as novel. In addition to a large number of isolates that are likely *B. cinerea*, *B. paeoniae*, or *B. pseudocinerea*, twenty seven (15%) of the isolates collected do not appear to fall into clades with any currently named *Botrytis* species based on molecular phylogenies and represent as many as 10 novel, distinct phylogenetic lineages. In this way, the *Botrytis* isolates collected from peonies in the Pacific Northwest could represent up to one-third of the total diversity currently described within the genus, including numerous potentially novel species. Although we have observed and reported their growth characteristics, definite determination of the taxonomic placement of many of these isolates would require additional morphological and phenotypical characterization, therefore, the species status of these isolates remains inconclusive. Nonetheless, genetic analysis supports their distinction, and similarities can be seen among isolates representing phylogenetic clades, such as those observed with the stellate growth pattern for isolates MS04, FH04, and NP19 on MEA. These three isolates also showed tight similarities in growth in the temperature trials. The temperature trials also revealed notable similarities between AP04 and AP05 which compose their own phylogenetic clade (AKBot8) and appear to have a lower optimum growth temperature and a lower temperature threshold at which no growth occurs as compared with all other isolates tested. These two isolates also shared the ability to consistently cause disease on peony in the pathogenicity trials. Among other phylogenetic groups, such as the group of 8 isolates designated at AKBot4, greater differences in cultural characteristics and growth rates were observed. This diversity, however, is not surprising and likely does not negate phylogenetic evidence of relatedness; it has been well documented that individuals within *Botrytis* species display great morphological dissimilarity and plasticity^16^. The isolates’ consistent abilities to cause disease in pathogenicity trials and apparent death following incubation at 35 C lend further credence to their representation as a single species. Although colony characteristics and growth data were generated, direct comparisons with named species cannot be made due to differences in cultural conditions of this study versus those under which previous species descriptions were made. Further analysis, including side-by-side growth trials, additional pathogenicity trials, and measurements of conidia, conidiophores, and sclerotia, is necessary to confirm whether or not the isolates collected in this study are in fact members of presently recognized species, such as *B. prunorum* and *B. fragariae*.

The results of the pathogenicity trials were quite variable and could be indicative of a wide range of factors that determine the ability of *Botrytis* species isolates to cause disease on peonies such as: differences in crop susceptibility at distinct phenological stages during the growing season, differences in resistance among peony cultivars, differences in susceptibility among plant parts, and the inability of the isolates to consistently cause disease under the conditions tested. The fact that isolates FH04, MS04, and NP19 (AKBot3) consistently caused lesions on the cultivar ‘Sarah Bernhardt’ in the first trial, but failed to cause disease in a repeat trial (executed 4 months later), suggests that phenology of the host could be an important factor in virulence. The same differences were evident among the first and second trials for GBG03 and GBG57 (AKBot9) on ‘Kansas’ (executed one month apart from each other). Differences in the ability to cause lesions within the same trial could also be due to differences in the leaves used as replicates. Limited information is available relating to leaf-to-leaf variation and changes in the susceptibility of peony foliage during the growing season, therefore these hypotheses are speculative. Although isolates GBG03 and GBG57 caused disease on ‘Kansas,’ they were not observed as causing disease as frequently in the trials on the cultivar ‘Sarah Bernhardt,’ which may indicate differences in cultivar susceptibility. Trials with *B. paeoniae*^38^ and our personal observations in the field have shown clear differences among the abilities of *Botrytis* to cause disease on different peony cultivars. Unfortunately, cultivar information was not collected for all isolates in our study to provide for adequate comparison. However, it is worth noting that many of the most commonly grown cultivars in Alaska are similar to those from Washington and Oregon, and in fact, planting material is regularly sourced from the latter two states. Plant part could also play a factor in the variable symptom development. Given that the isolates were collected from various above-ground plant parts, but only tested on foliage, it is possible that the isolates are indeed pathogenic on peonies, but unable to consistently cause disease on foliage. This may be the case for isolates collected from flower or flower bud decay given the high susceptibility of flower tissues to *Botrytis*. The possibility remains also that not all the species found in this survey are pathogens of peony, instead, could be acting as saprophytes like the recently described *B. pyriformis* found associated with the plant species *Sedum sarmentosum*^10^. This could be especially true for isolates collected from blasted buds or leaf tip dieback which may have become necrotic prior to colonization by *Botrytis* (see Supplementary Figs S12–S20 for symptom descriptions and example photos). Lastly, the variability in disease among trials could be an indication that the testing conditions were not ideal for consistent disease development for some of the isolates. Additional testing would be required to find out ideal infection conditions for these putatively novel *Botrytis* species.

Although the pathogenicity of the isolates found in this study on peony are still somewhat unclear at this time, this survey represents the most *Botrytis* diversity reported from a single host plant, despite recent efforts to explore diversity in other cropping systems. Recent surveys on crops including onion, bean (*Phaseolous*), eucalyptus (*Eucalyptus*), and *Sedum* in China^10,16,21,22^, grape in the United States and China^18,23^, strawberry in Germany^17^, plum in Chile^11^, and blackberry (*Rubus* spp.) in the United States^15^ have all revealed new *Botrytis* species, but without the great diversity observed in the present survey. Traditionally, *Allium* species have had the most reported diversity of *Botrytis* species of any host crop; 7 *Botrytis* species and 1 hybrid species have been reported on *Allium*^1^. The present survey indicates that peonies may be host to more than twice the number of *Botrytis* species as currently described on *Allium*. Future exploration of other cropping systems may reveal similar diversity seen on peonies in this survey, however, no reports of this nature currently exist. For growers in Alaska, how the diversity of *Botrytis* in peonies will affect their disease management programs is unclear. A clearer understanding of the biology, epidemiology and fungicide sensitivity of these *Botrytis*, along with an understanding of potential variation in pathogenicity and host resistance to pathogenic species, is needed to determine what if any changes are needed in current disease management recommendations.

The results of the *HSP60* analysis with the endophytic isolates from *C. stoebe* and *T. officinale* should be interpreted with caution considering the short sequence length of the *C. stoebe* and *T. officinale Botrytis* isolates; some of the sequence that is missing may contain phylogenetically informative polymorphisms within genus *Botrytis*^6^. Nonetheless, the phylogenetic analysis suggests that some of the *Botrytis* species found in Alaska may also behave as endophytes in *C. stoebe*. These findings beg the question of whether or not some of the fungi infecting peonies behave as endophytes in peonies or in surrounding host plants in Alaska. While this hypothesis is highly speculative, recent research has clearly demonstrated the ability of certain *Botrytis* species to exist as symptomless endophytes while maintaining virulence and the ability to sporulate on host tissue^7,32,35,36,39^. It is worth noting that *C. stoebe* has been reported as an invasive species in Alaska, however, the range of this species is minimal in the state and efforts to contain its spread have been largely successful^40^. Studies to identify endophytes in native Alaskan species may yield additional insight into the ecological role of the *Botrytis* species found on peony in this survey.

Some of the isolates from peony were phylogenetically similar to *Botrytis* species recently discovered on plum and kiwi (*Actinidia deliciosa*) (*B. prunorum*)^11,41^ and strawberry (*B. fragariae*)^17^. These results, considered together with other reports of newly-described *Botrytis* species on multiple host crops^18^, suggest that there may be a higher rate of polyphagy in genus *Botrytis* than previous thought^6^. The results of this survey also suggest a wide geographical and climactic range for some *Botrytis* species, as the aforementioned species were found in Chile (*B. prunorum*)^11,41^ and Germany (*B. fragariae*)^17^, respectively. The exact sampling locations of the *C. stoebe* endophytic isolates are unclear, however, none were collected in Alaska^31^. Further studies on the biology of these *Botrytis* species would help to elucidate the apparent potential for *Botrytis* species to colonize (either as a pathogen, saprophyte, or endophyte) multiple hosts in diverse climates.

It’s unclear why so much genetic diversity was present in the surveys that took place during this study as compared with the numerous other efforts worldwide to characterize *Botrytis* species in other cropping systems. Given that the putatively new species isolates found in this study are able to grow on BSM, it is not likely that the use of this media is a contributor of the lower yield in species diversity in other studies. The fact that the highest amount of diversity was found in Alaska as compared with Washington and Oregon, especially given their similarity in popular cultivars and trade in planting material, suggests that the high diversity may be related to sampling location rather than host species, yet without controlled studies this hypothesis remains speculative. Future surveys of both pathogens and endophytes of other crops and native plant species in Alaska and other boreal forest regions may help elucidate the host range, biology, and ecological roles of the *Botrytis* species found during this survey.

This study represents a relatively small sampling of *Botrytis* species on peony, yet revealed a large amount of genetic diversity. Additional surveys, especially in Alaska, may reveal an even greater amount of diversity given that small sample sizes are likely to underestimate total diversity. Taken as a whole, this survey indicates that the scientific understanding of *Botrytis* species diversity, geographic distribution, host-specificity, and their potential ecological roles as pathogens, saprophytes, or endophytes is indeed in its infancy and there is likely much more to be learned about this economically- and scientifically-important genus.

## Materials and Methods

### Surveys, collections, and isolations

Peony (*Paeonia lactiflora* cultivars) fields were sampled to identify plants infected with *Botrytis* over the course of the 2014–2015 growing seasons in Alaska, Washington, and Oregon. A randomized field sampling strategy was not employed, however attempts were made to collect a range of symptoms present in a single field, including acquiring samples from a range of cultivars, above-ground plant tissues, and lesion shapes, sizes, and colours, and necrotic tissues such as blasted buds. Sampling was focused on collecting tissues displaying characteristic symptoms of *Botrytis* infection; samples were not taken from asymptomatic tissue. Photographs of representative samples showing the various symptomatic tissues collected during the surveys are given in Supplementary Figs S12–S20. Samples were also acquired from growers by mail during the research period. A total of 23 fields in Alaska, 8 fields in Washington, and 4 fields in Oregon were represented in the survey. Four fields in Alaska, 2 fields in Washington, and one in Oregon were surveyed in multiple years. All but three fields surveyed (one at a botanical garden in Fairbanks, AK, one at a research facility in Puyallup, WA, and one at a research facility in Mt. Vernon, WA) were commercial peony fields. Maps of the approximate survey areas in each state are provided in Supplementary Fig. S21 and a complete list of the isolates collected is provided in Supplementary Table S1.

Isolations were performed to recover the fungal pathogen from plant samples. A small piece of plant material from the margin between symptomatic and healthy tissue was excised and surface sterilized in a 1% NaOCl solution for 30 seconds, rinsed twice in sterile water, and plated onto potato dextrose agar amended with streptomycin and chloramphenicol (PDA + s/c) as described by Garfinkel *et al*.^13^. Subsequent fungal growth was transferred and hyphal-tipped to ensure only one organism was isolated in culture for further analysis.

### DNA extraction, PCR and sequencing

DNA extractions, PCR, and sequencing were performed as described by Garfinkel *et al*.^13^. Pure, hyphal-tipped cultures were transferred onto PDA + s/c overlaid with sterile cellophane membrane, scraped into sterile 2.0 mL Eppendorf tubes, and frozen at −80 C. Samples were homogenized with the aid of sterile glass beads and DNA was extracted from the homogenized mycelium as per the manufacturer’s instructions using a DNA extraction kit (Gentra Puregene Tissue Kit, Qiagen, Redwood City, California).

PCR was used to amplify the *G3PDH* gene from a total of 178 isolates from peony (Supplementary Table S1) using primers from Staats *et al*.^6^ or slightly modified primers which were synthesized without M-13 universal primer tags^13^. PCR reactions and cycling conditions were as described in Garfinkel *et al*.^13^. The *HSP60*, *RPB2*^6^, *NEP1*, and *NEP2* genes^24^ were also amplified for a subset of isolates (Supplementary Table S1) using primers, reagents, and cycling conditions as described in Garfinkel *et al*.^13^. Resulting PCR products were cleaned up and then sequenced using the same primers used in amplification. Some PCR products were sequenced in both directions to get adequate sequence length for phylogenetic analysis and to confirm sequence polymorphisms or ambiguities. Those products not sequenced in both directions were deemed to have adequate sequence length and base calls of sufficient quality for analysis that a reverse sequence was not needed to supplement forward sequence data.

### Construction of phylogenetic trees

For those products that were sequenced in forward and reverse, consensus sequences were constructed using Geneious (v. 8.1.4)^42^. Sequences of *Botrytis* from peony were aligned with sequences of *Botrytis* spp. isolates and *Sclerotinia sclerotiorum* as an outgroup. Sequences used in analysis were either retrieved from GenBank, built from publicly available whole genome sequence data, or consisted of unpublished sequence data developed for this study or shared from other labs. A full list of the sequences used in the phylogenetic analysis and their sources can be found in Supplementary Table S2. Alignments were estimated using Clustal W implemented in MEGA (v. 6.06)^43^ using the default parameters as described by Zhang *et al*.^21^ and Garfinkel *et al*.^13^. All sequences within a gene were trimmed to equal length, except for the sequences for *B. fragariae*^17^, *B. mali*^44^, *B. sinoviticola*^23^, and *B. euroamericana* isolate B83^13,37^ which were often shorter (up to 300 bp) than those of the other isolates.

Individual gene trees were constructed for the *G3PDH*, *HSP60*, *RPB2*, *HSP60*, *NEP1*, and *NEP2* gene trees using both maximum likelihood (ML)^45,46^ and neighbour joining (NJ)^47^ methods. Trees were visually assessed and it was observed that the same isolates consistently fell into distinct, well-supported clades among all genes. Given the similarity in groupings between the *G3PDH*, *HSP60*, *RPB2*, *NEP1* and *NEP2* individual gene trees, these gene sequences were concatenated to produce a combined gene tree. Individual and combined ML gene trees for all genes were constructed using nucleotide substitution models selected using the Bayesian information criterion (BIC) generated in MEGA. Individual models are specified for each gene tree in the figure captions (Figs 1, 2, Supplementary Figs S1–S5). Gaps and missing data were partially deleted using a 95% site coverage cutoff. All trees were inferred with 1000 bootstrap replicates.

The *Botrytis* isolates from peony were also aligned with *HSP60* sequences of currently recognized *Botrytis* spp. and unidentified endophytic *Botrytis* spp. isolated from the invasive plant species *Centaurea stoebe*^31^ and *Taxacum officinale*^32^, using *S. sclerotium* as an outgroup (Supplementary Table S2). Sequence alignment parameters were the same as described above, however, due to the short sequence length of some of the *C. stoebe*^31^ and *Taxacum officinale*^32^ sequences, not all sequences were trimmed to identical length. Phylogenetic analysis was therefore conducted with the endophytic *Botrytis* sequences being, in some cases, much shorter than the reference *Botrytis* isolates, the outgroup sequence, and the isolates from peony. Maximum likelihood nucleotide substitution models were selected using the BIC. All trees were constructed with 1000 bootstrap replicates.

An analysis of the *G3PDH* + *HSP60* genes combined was used to compare the *Botrytis* isolates from peony identified as *B. pseudocinerea* (Fig. 1) to additional *B. pseudocinerea* isolates from wine grapes in New Zealand and France and relevant outgroups (Supplementary Table S2). Peony *Botrytis* species isolates AP04 and AP05 were also included in the analysis to determine if they were representatives of this species. All sequence alignment and tree construction parameters were the same as described above.

All alignments and trees were deposited into TreeBase for public access (http://purl.org/phylo/treebase/phylows/study/TB2:S22397).

### Cultural characterization

Plugs (5 mm diameter) were cut from the actively growing margin of colonies and transferred to MEA, PDA, or BSM^34^, mycelium side-down, and incubated either in the dark or under constant UV light (350 nm) at 20 C; isolates on BSM were only incubated in the dark. UV light was selected for growth trials given its ability to promote sporulation of *B. cinerea*^48^. Isolates were transferred in triplicates to each light-media combination. Isolates were removed from the incubators, photographed, and observed to record colony morphology and evidence of sporulation and sclerotia development at 4, 7, 14, and 21 days and placed back into the same incubation conditions. In addition to the 31 putatively new *Botrytis* species isolates identified in the present study, *B. cinerea* isolate MS05^13^ and *B. paeoniae* isolate AR05^49^, previously isolated from peony, were grown as a comparison to assess relative levels of growth and sporulation.

### Growth rate and temperature trials

Plugs (5 mm diameter) were cut from the edges of actively growing colonies of *Botrytis* isolates grown up on PDA at 20 C in the dark and transferred to the centre of new PDA petri plates, mycelium side-down. Three replicates of each isolate were placed in growth chambers set at 5, 10, 15, 20, 25, 30, and 35 C and incubated in the dark. At 48 hours, two perpendicular measurements were taken to calculate average colony diameter. The 4 mm plug was subtracted from reported colony diameter measurements. Trials were repeated twice and data from the two trials were combined for a total of 6 replicates per isolate. An analysis of variance was performed to determine statistical differences among diameters at different temperatures within an isolate. Post-hoc means separations were done using Tukey’s range test at α = 0.05.

### Pathogenicity trials

Healthy appearing and fully expanded peony foliage from the cultivars ‘Kansas’ and ‘Sarah Bernhardt’ was harvested from the middle portion of the stems of whole peony plants. Leaflets were cut along the lobes to a size that would fit into a 15 cm diameter petri plate and surface sterilized in a 10% bleach solution for 30–60 seconds and then rinsed twice in sterile deionized water. Leaf pieces were placed individually into sterile petri plates that contained sterile filter papers moistened with sterile water. Leaflets for the repeat test was taken from a second set of plants that had emerged at the same time as the first round; care was taken to harvest foliage from the same location on the plant. Due to possible variation among leaves, for each round of tests, leaflet pieces were randomly assigned to be inoculated with each *Botrytis* species isolate and a control treatment. A 5 mm plug was cut from the edge of actively growing colonies of *Botrytis* isolates that had been grown up on PDA at 20 C in the dark and plugs were placed mycelium side-down on the abaxial side of the leaf surface. Plates were stored in a plastic bin containing wet paper towels and the bin was wrapped in a plastic bag to maintain high humidity conditions. Leaves were incubated at 20 C in the dark and observed every 48 hours for lesion development. At 4, 6, and 7 days post inoculation, two perpendicular measurements of resulting lesion diameters were taken and an average lesion diameter was calculated. Three leaves were inoculated per isolate per cultivar. All trials were performed twice and used known peony pathogens *B. cinerea* and *B. paeoniae* as controls.

## Supplementary information

All Supplementary Data Merged
