# Supplementary material for: Genetic analysis reveals unprecedented diversity of a globally-important plant pathogenic genus
Source: Sci Rep. 2019 Apr 30;9:6671. doi: 10.1038/s41598-019-43165-y (PMC6491473; doi:10.1038/s41598-019-43165-y)
Supplement: Supplementary file 1 — All Supplementary Data Merged [file 41598_2019_43165_MOESM1_ESM.pdf]

Supplementary Information

Genetic analysis reveals unprecedented diversity of a globally-important plant pathogenic genus

Andrea R. Garfinkel*, Katie P. Coats, Don L. Sherry, and Gary A. Chastagner
Washington State University Puyallup Research and Extension Center, 2606 W. Pioneer, Puyallup, WA, 98371
Email: andrea.garfinkel@wsu.edu

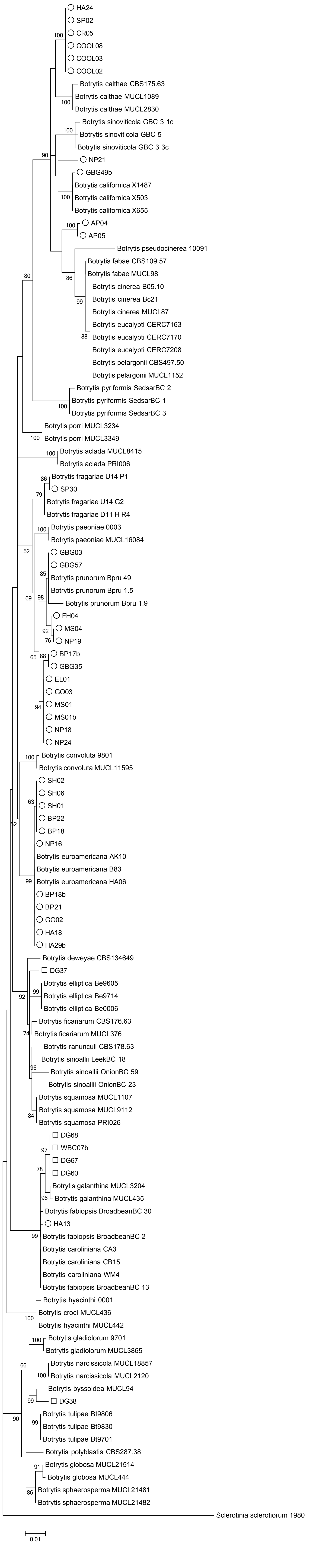
HA24
SP02
CR05
COOL08
COOL03
COOL02
Botrytis calthae CBS175.63
Botrytis calthae MUCL1089
Botrytis calthae MUCL2830
Botrytis sinoviticola GBC 3 1c
Botrytis sinoviticola GBC 5
Botrytis sinoviticola GBC 3 3c
NP21
GBG49b
Botrytis californica X1487
Botrytis californica X503
Botrytis californica X655
AP04
AP05
Botrytis pseudocinerea 10091
Botrytis fabae CBS109.57
Botrytis fabae MUCL98
Botrytis cinerea B05.10
Botrytis cinerea Bc21
Botrytis cinerea MUCL87
Botrytis eucalypti CERC7163
Botrytis eucalypti CERC7170
Botrytis eucalypti CERC7208
Botrytis pelargonii CBS497.50
Botrytis pelargonii MUCL1152
Botrytis pyriformis SedsarBC 2
Botrytis pyriformis SedsarBC 1
Botrytis pyriformis SedsarBC 3
Botrytis porri MUCL3234
Botrytis porri MUCL3349
Botrytis aclada MUCL8415
Botrytis aclada PRI006
Botrytis fragariae U14 P1
SP30
Botrytis fragariae U14 G2
Botrytis fragariae D11 H R4
Botrytis paeoniae 0003
Botrytis paeoniae MUCL16084
GBG03
GBG57
Botrytis prunorum Bpru 49
Botrytis prunorum Bpru 1.5
Botrytis prunorum Bpru 1.9
FH04
MS04
NP19
BP17b
GBG35
EL01
GO03
MS01
MS01b
NP18
NP24
Botrytis convoluta 9801
Botrytis convoluta MUCL11595
SH02
SH06
SH01
BP22
BP18
NP16
Botrytis euroamericana AK10
Botrytis euroamericana B83
Botrytis euroamericana HA06
BP18b
BP21
GO02
HA18
HA29b
Botrytis deweyae CBS134649
DG37
Botrytis elliptica Be9605
Botrytis elliptica Be9714
Botrytis elliptica Be0006
Botrytis ficariarum CBS176.63
Botrytis ficariarum MUCL376
Botrytis ranunculi CBS178.63
Botrytis sinoallii LeekBC 18
Botrytis sinoallii OnionBC 59
Botrytis sinoallii OnionBC 23
Botrytis squamosa MUCL1107
Botrytis squamosa MUCL9112
Botrytis squamosa PRI026
DG68
WBC07b
DG67
DG60
Botrytis galanthina MUCL3204
Botrytis galanthina MUCL435
Botrytis fabiopsis BroadbeanBC 30
HA13
Botrytis fabiopsis BroadbeanBC 2
Botrytis caroliniana CA3
Botrytis caroliniana CB15
Botrytis caroliniana WM4
Botrytis fabiopsis BroadbeanBC 13
Botrytis hyacinthi 0001
Botrytis croci MUCL436
Botrytis hyacinthi MUCL442
Botrytis gladiolorum 9701
Botrytis gladiolorum MUCL3865
Botrytis narcissicola MUCL18857
Botrytis narcissicola MUCL2120
Botrytis byssoidea MUCL94
DG38
Botrytis tulipae Bt9806
Botrytis tulipae Bt9830
Botrytis tulipae Bt9701
Botrytis polyblastis CBS287.38
Botrytis globosa MUCL21514
Botrytis globosa MUCL444
Botrytis sphaerosperma MUCL21481
Botrytis sphaerosperma MUCL21482
Sclerotinia sclerotiorum 1980
0.01

Supplementary Fig. S1 Maximum likelihood tree of *G3PDH* gene sequences. The tree describes the relationship of *Botrytis* species isolates collected from peonies in Alaska (indicated by ○) and Washington (indicated by □) to named *Botrytis* species using *Sclerotinia sclerotiorum* as an outgroup. A total of 871 positions were used in the final dataset. Evolutionary relationships were modeled using a Tamura-Nei model with gamma distribution rates and invariable sites. Bootstrap percentages (n=1000) are shown on branches. Branches with <50% bootstrap support are not shown. The tree is drawn to scale with branch lengths proportional to the number of substitutions per site.

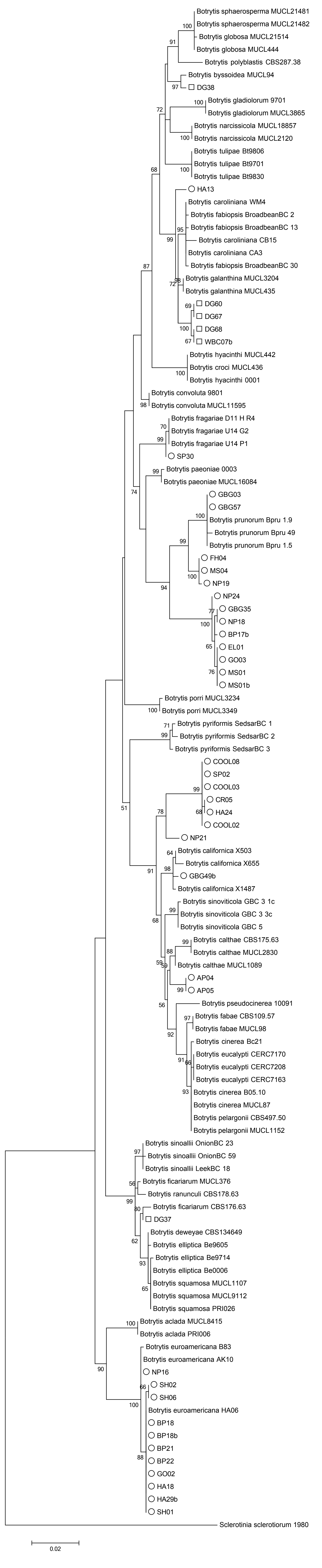
Botrytis sphaerosperma MUCL21481
Botrytis sphaerosperma MUCL21482
Botrytis globosa MUCL21514
Botrytis globosa MUCL444
Botrytis polyblastis CBS287.38
Botrytis byssoidea MUCL94
DG38
Botrytis gladiolorum 9701
Botrytis gladiolorum MUCL3865
Botrytis narcissicola MUCL18857
Botrytis narcissicola MUCL2120
Botrytis tulipae Bt9806
Botrytis tulipae Bt9701
Botrytis tulipae Bt9830
HA13
Botrytis caroliniana WM4
Botrytis fabiopsis BroadbeanBC 2
Botrytis fabiopsis BroadbeanBC 13
Botrytis caroliniana CB15
Botrytis caroliniana CA3
Botrytis fabiopsis BroadbeanBC 30
Botrytis galanthina MUCL3204
Botrytis galanthina MUCL435
DG60
DG67
DG68
WBC07b
Botrytis hyacinthi MUCL442
Botrytis croci MUCL436
Botrytis hyacinthi 0001
Botrytis convoluta 9801
Botrytis convoluta MUCL11595
Botrytis fragariae D11 H R4
Botrytis fragariae U14 G2
Botrytis fragariae U14 P1
SP30
Botrytis paeoniae 0003
Botrytis paeoniae MUCL16084
GBG03
GBG57
Botrytis prunorum Bpru 1.9
Botrytis prunorum Bpru 49
Botrytis prunorum Bpru 1.5
FH04
MS04
NP19
NP24
GBG35
NP18
BP17b
EL01
GO03
MS01
MS01b
Botrytis porri MUCL3234
Botrytis porri MUCL3349
Botrytis pyriformis SedsarBC 1
Botrytis pyriformis SedsarBC 2
Botrytis pyriformis SedsarBC 3
COOL08
SP02
COOL03
CR05
HA24
COOL02
NP21
Botrytis californica X503
Botrytis californica X655
GBG49b
Botrytis californica X1487
Botrytis sinoviticola GBC 3 1c
Botrytis sinoviticola GBC 3 3c
Botrytis sinoviticola GBC 5
Botrytis calthae CBS175.63
Botrytis calthae MUCL2830
Botrytis calthae MUCL1089
AP04
AP05
Botrytis pseudocinerea 10091
Botrytis fabae CBS109.57
Botrytis fabae MUCL98
Botrytis cinerea Bc21
Botrytis eucalypti CERC7170
Botrytis eucalypti CERC7208
Botrytis eucalypti CERC7163
Botrytis cinerea B05.10
Botrytis cinerea MUCL87
Botrytis pelargonii CBS497.50
Botrytis pelargonii MUCL1152
Botrytis sinoallii OnionBC 23
Botrytis sinoallii OnionBC 59
Botrytis sinoallii LeekBC 18
Botrytis ficariarum MUCL376
Botrytis ranunculi CBS178.63
Botrytis ficariarum CBS176.63
DG37
Botrytis deweyae CBS134649
Botrytis elliptica Be9605
Botrytis elliptica Be9714
Botrytis elliptica Be0006
Botrytis squamosa MUCL1107
Botrytis squamosa MUCL9112
Botrytis squamosa PRI026
Botrytis aclada MUCL8415
Botrytis aclada PRI006
Botrytis euroamericana B83
Botrytis euroamericana AK10
NP16
SH02
SH06
Botrytis euroamericana HA06
BP18
BP18b
BP21
BP22
GO02
HA18
HA29b
SH01
Sclerotinia sclerotiorum 1980
0.02

Supplementary Fig. S2 Maximum likelihood tree of *HSP60* gene sequences. The tree describes the relationship of *Botrytis* species isolates collected from peonies in Alaska (indicated by ○) and Washington (indicated by □) to named *Botrytis* species using *Sclerotinia sclerotiorum* as an outgroup. A total of 834 positions were used in the final dataset. Evolutionary relationships were modeled using a Kimura 2-parameter model with gamma distribution rates. Bootstrap percentages (n=1000) are shown on branches. Branches with <50% bootstrap support are not shown. The tree is drawn to scale with branch lengths proportional to the number of substitutions per site.

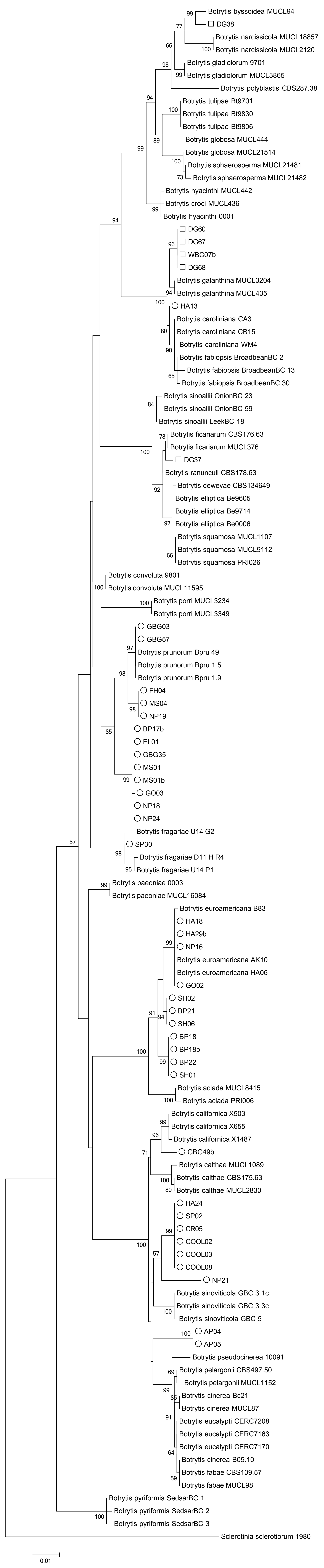
Botrytis byssoidea MUCL94
DG38
Botrytis narcissicola MUCL18857
Botrytis narcissicola MUCL2120
Botrytis gladiolorum 9701
Botrytis gladiolorum MUCL3865
Botrytis polyblastis CBS287.38
Botrytis tulipae Bt9701
Botrytis tulipae Bt9830
Botrytis tulipae Bt9806
Botrytis globosa MUCL444
Botrytis globosa MUCL21514
Botrytis sphaerosperma MUCL21481
Botrytis sphaerosperma MUCL21482
Botrytis hyacinthi MUCL442
Botrytis croci MUCL436
Botrytis hyacinthi 0001
DG60
DG67
WBC07b
DG68
Botrytis galanthina MUCL3204
Botrytis galanthina MUCL435
HA13
Botrytis caroliniana CA3
Botrytis caroliniana CB15
Botrytis caroliniana WM4
Botrytis fabiopsis BroadbeanBC 2
Botrytis fabiopsis BroadbeanBC 13
Botrytis fabiopsis BroadbeanBC 30
Botrytis sinoallii OnionBC 23
Botrytis sinoallii OnionBC 59
Botrytis sinoallii LeekBC 18
Botrytis ficariarum CBS176.63
Botrytis ficariarum MUCL376
DG37
Botrytis ranunculi CBS178.63
Botrytis deweyae CBS134649
Botrytis elliptica Be9605
Botrytis elliptica Be9714
Botrytis elliptica Be0006
Botrytis squamosa MUCL1107
Botrytis squamosa MUCL9112
Botrytis squamosa PRI026
Botrytis convoluta 9801
Botrytis convoluta MUCL11595
Botrytis porri MUCL3234
Botrytis porri MUCL3349
GBG03
GBG57
Botrytis prunorum Bpru 49
Botrytis prunorum Bpru 1.5
Botrytis prunorum Bpru 1.9
FH04
MS04
NP19
BP17b
EL01
GBG35
MS01
MS01b
GO03
NP18
NP24
Botrytis fragariae U14 G2
SP30
Botrytis fragariae D11 H R4
Botrytis fragariae U14 P1
Botrytis paeoniae 0003
Botrytis paeoniae MUCL16084
Botrytis euroamericana B83
HA18
HA29b
NP16
Botrytis euroamericana AK10
Botrytis euroamericana HA06
GO02
SH02
BP21
SH06
BP18
BP18b
BP22
SH01
Botrytis aclada MUCL8415
Botrytis aclada PRI006
Botrytis californica X503
Botrytis californica X655
Botrytis californica X1487
GBG49b
Botrytis calthae MUCL1089
Botrytis calthae CBS175.63
Botrytis calthae MUCL2830
HA24
SP02
CR05
COOL02
COOL03
COOL08
NP21
Botrytis sinoviticola GBC 3 1c
Botrytis sinoviticola GBC 3 3c
Botrytis sinoviticola GBC 5
AP04
AP05
Botrytis pseudocinerea 10091
Botrytis pelargonii CBS497.50
Botrytis pelargonii MUCL1152
Botrytis cinerea Bc21
Botrytis cinerea MUCL87
Botrytis eucalypti CERC7208
Botrytis eucalypti CERC7163
Botrytis eucalypti CERC7170
Botrytis cinerea B05.10
Botrytis fabae CBS109.57
Botrytis fabae MUCL98
Botrytis pyriformis SedsarBC 1
Botrytis pyriformis SedsarBC 2
Botrytis pyriformis SedsarBC 3
Sclerotinia sclerotiorum 1980
0.01

Supplementary Fig. S3 Maximum likelihood tree of *RPB2* gene sequences. The tree describes the relationship of *Botrytis* species isolates collected from peonies in Alaska (indicated by ○) and Washington (indicated by □) to named *Botrytis* species using *Sclerotinia sclerotiorum* as an outgroup. A total of 1092 positions were used in the final dataset. Evolutionary relationships were modeled using a Tamura 3-parameter model with gamma distribution rates. Bootstrap percentages (n=1000) are shown on branches. Branches with <50% bootstrap support are not shown. The tree is drawn to scale with branch lengths proportional to the number of substitutions per site.

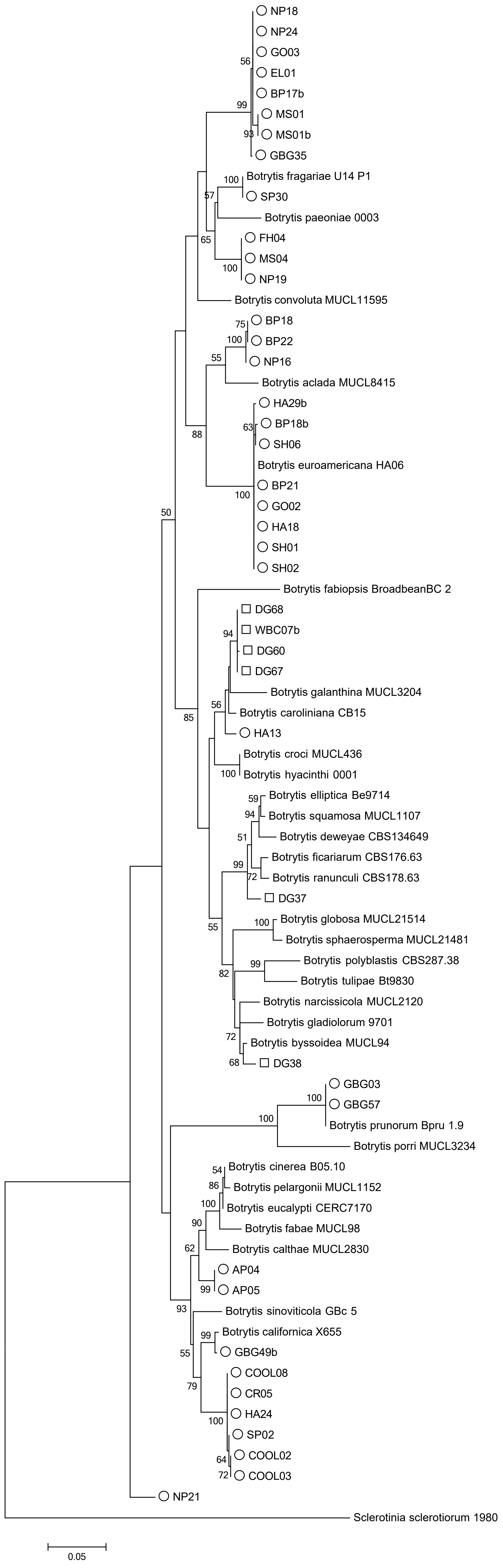

NP18
NP24
GO03
EL01
BP17b
MS01
MS01b
GBG35
Botrytis fragariae U14 P1
SP30
Botrytis paeoniae 0003
FH04
MS04
NP19
Botrytis convoluta MUCL11595
BP18
BP22
NP16
Botrytis aclada MUCL8415
HA29b
BP18b
SH06
Botrytis euroamericana HA06
BP21
GO02
HA18
SH01
SH02
Botrytis fabiopsis BroadbeanBC 2
DG68
WBC07b
DG60
DG67
Botrytis galanthina MUCL3204
Botrytis caroliniana CB15
HA13
Botrytis croci MUCL436
Botrytis hyacinthi 0001
Botrytis elliptica Be9714
Botrytis squamosa MUCL1107
Botrytis deweyae CBS134649
Botrytis ficariarum CBS176.63
Botrytis ranunculi CBS178.63
DG37
Botrytis globosa MUCL21514
Botrytis sphaerosperma MUCL21481
Botrytis polyblastis CBS287.38
Botrytis tulipae Bt9830
Botrytis narcissicola MUCL2120
Botrytis gladiolorum 9701
Botrytis byssoidea MUCL94
DG38
GBG03
GBG57
Botrytis prunorum Bpru 1.9
Botrytis porri MUCL3234
Botrytis cinerea B05.10
Botrytis pelargonii MUCL1152
Botrytis eucalypti CERC7170
Botrytis fabae MUCL98
Botrytis calthae MUCL2830
AP04
AP05
Botrytis sinoviticola GBc 5
Botrytis californica X655
GBG49b
COOL08
CR05
HA24
SP02
COOL02
COOL03
NP21
Sclerotinia sclerotiorum 1980
0.05

Supplementary Fig. S4 Maximum likelihood tree of *NEP1* gene sequences. The tree describes the relationship of *Botrytis* species isolates collected from peonies in Alaska (indicated by ○) and Washington (indicated by □) to named *Botrytis* species using *Sclerotinia sclerotiorum* as an outgroup. A total of 678 positions were used in the final dataset. Evolutionary relationships were modeled using a Kimura 2-parameter model with gamma distribution rates. Bootstrap percentages (n=1000) are shown on branches. Branches with <50% bootstrap support are not shown. The tree is drawn to scale with branch lengths proportional to the number of substitutions per site.

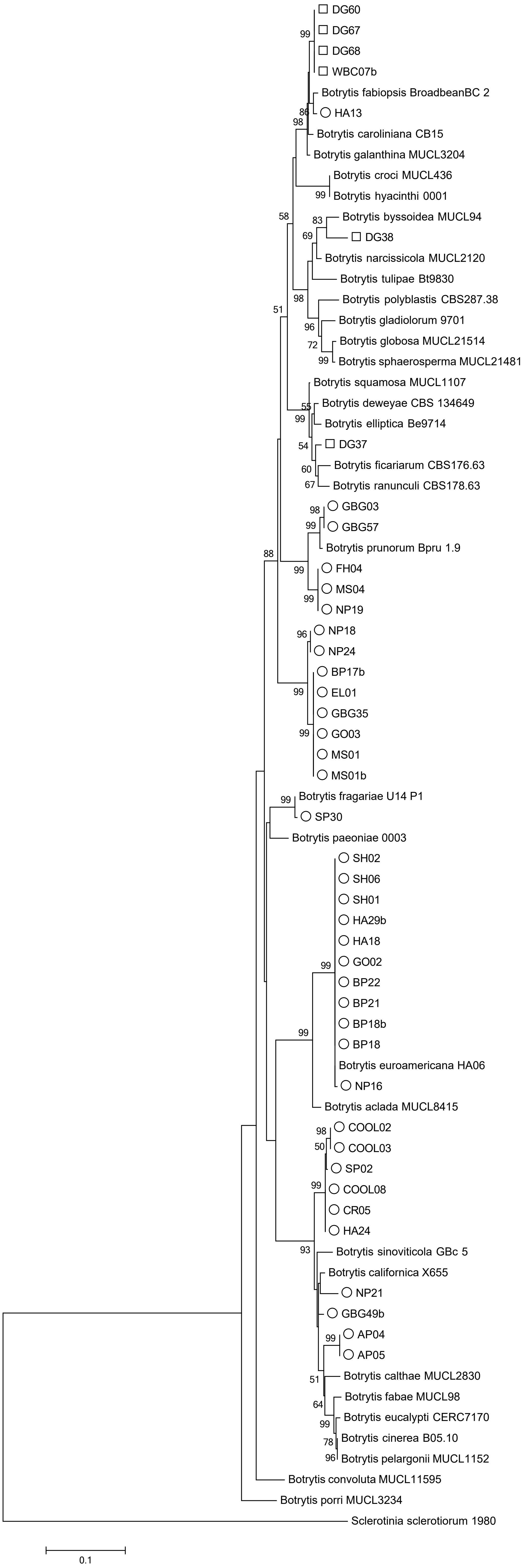

DG60
DG67
DG68
WBC07b
Botrytis fabiopsis BroadbeanBC 2
HA13
Botrytis caroliniana CB15
Botrytis galanthina MUCL3204
Botrytis croci MUCL436
Botrytis hyacinthi 0001
Botrytis byssoidea MUCL94
DG38
Botrytis narcissicola MUCL2120
Botrytis tulipae Bt9830
Botrytis polyblastis CBS287.38
Botrytis gladiolorum 9701
Botrytis globosa MUCL21514
Botrytis sphaerosperma MUCL21481
Botrytis squamosa MUCL1107
Botrytis deweyae CBS 134649
Botrytis elliptica Be9714
DG37
Botrytis ficariarum CBS176.63
Botrytis ranunculi CBS178.63
GBG03
GBG57
Botrytis prunorum Bpru 1.9
FH04
MS04
NP19
NP18
NP24
BP17b
EL01
GBG35
GO03
MS01
MS01b
Botrytis fragariae U14 P1
SP30
Botrytis paeoniae 0003
SH02
SH06
SH01
HA29b
HA18
GO02
BP22
BP21
BP18b
BP18
Botrytis euroamericana HA06
NP16
Botrytis aclada MUCL8415
COOL02
COOL03
SP02
COOL08
CR05
HA24
Botrytis sinoviticola GBc 5
Botrytis californica X655
NP21
GBG49b
AP04
AP05
Botrytis calthae MUCL2830
Botrytis fabae MUCL98
Botrytis eucalypti CERC7170
Botrytis cinerea B05.10
Botrytis pelargonii MUCL1152
Botrytis convoluta MUCL11595
Botrytis porri MUCL3234
Sclerotinia sclerotiorum 1980
0.1

Supplementary Fig. S5 Maximum likelihood tree of *NEP2* gene sequences. The tree describes the relationship of *Botrytis* species isolates collected from peonies in Alaska (indicated by ○) and Washington (indicated by □) to named *Botrytis* species using *Sclerotinia sclerotiorum* as an outgroup. A total of 750 positions were used in the final dataset. Evolutionary relationships were modeled using a Kimura 2-parameter model with gamma distribution rates. Bootstrap percentages (n=1000) are shown on branches. Branches with <50% bootstrap support are not shown. The tree is drawn to scale with branch lengths proportional to the number of substitutions per site.

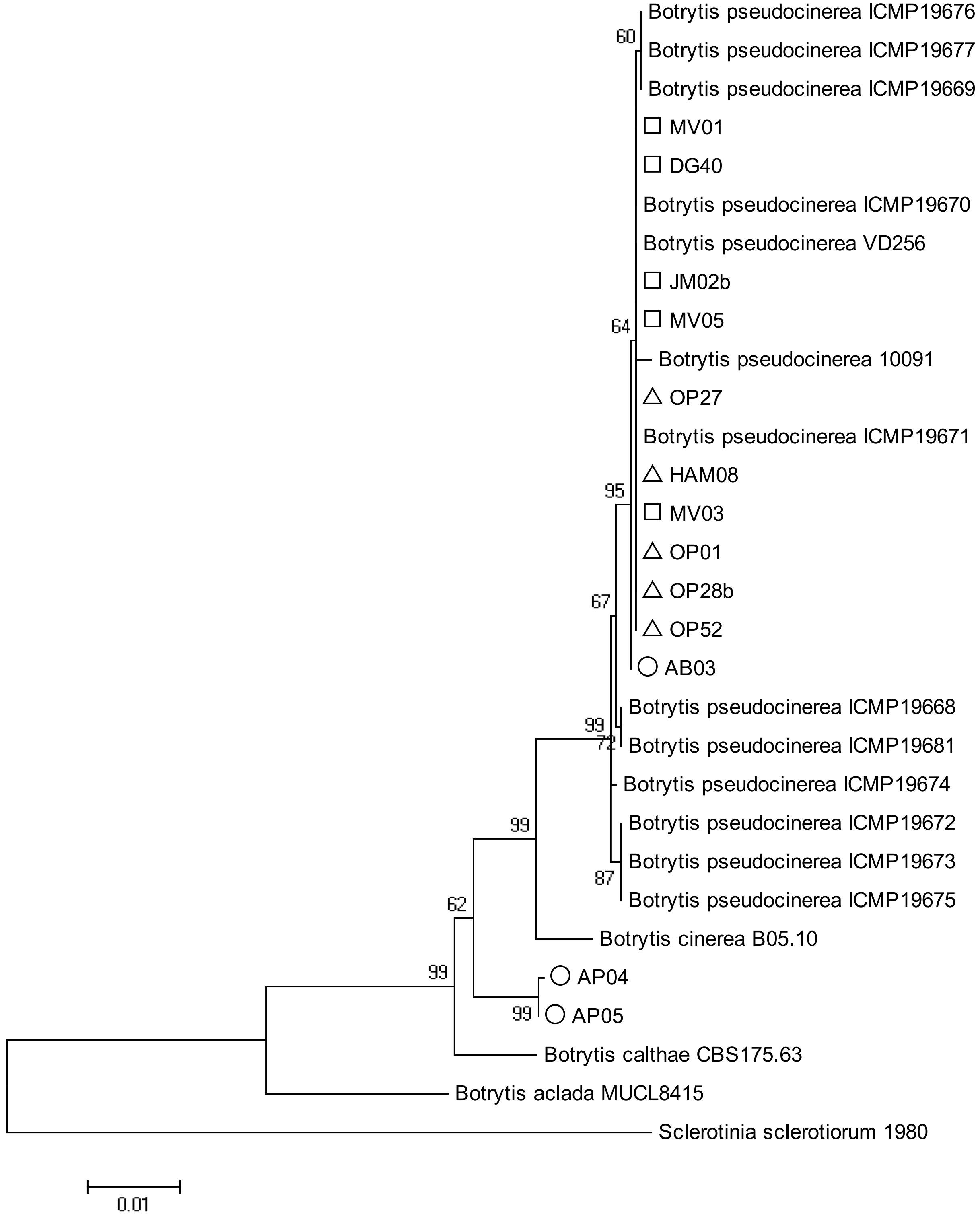

Botrytis pseudocinerea ICMP19676
Botrytis pseudocinerea ICMP19677
Botrytis pseudocinerea ICMP19669
MV01
DG40
Botrytis pseudocinerea ICMP19670
Botrytis pseudocinerea VD256
JM02b
MV05
Botrytis pseudocinerea 10091
OP27
Botrytis pseudocinerea ICMP19671
HAM08
MV03
OP01
OP28b
OP52
AB03
Botrytis pseudocinerea ICMP19668
Botrytis pseudocinerea ICMP19681
Botrytis pseudocinerea ICMP19674
Botrytis pseudocinerea ICMP19672
Botrytis pseudocinerea ICMP19673
Botrytis pseudocinerea ICMP19675
Botrytis cinerea B05.10
AP04
AP05
Botrytis calthae CBS175.63
Botrytis aclada MUCL8415
Sclerotinia sclerotiorum 1980
60
64
95
67
99
72
87
99
62
99
99
0.01

Supplementary Fig. S6 Maximum likelihood tree of combined *G3PDH+HSP60* gene sequences. The tree describes the relationship of 11 *Botrytis pseudocinerea* species isolates and two additional unnamed *Botrytis* sp. isolates (AP04 and AP05) collected from peonies in peonies in Alaska (indicated by ○), Oregon (indicated by Δ), and Washington (indicated by □) to reference *B. pseudocinerea* species isolates, *B. cinerea*, *B. calthae*, and *B. aclada*, using *Sclerotinia sclerotiorum* as an outgroup. A total of 1820 positions were used in the final dataset. Evolutionary relationships were modeled using a Tamura-Nei model with gamma distribution rates. Bootstrap percentages (n=1000) are shown on branches. Branches with <50% bootstrap support are not shown. The tree is drawn to scale with branch lengths proportional to the number of substitutions per site.

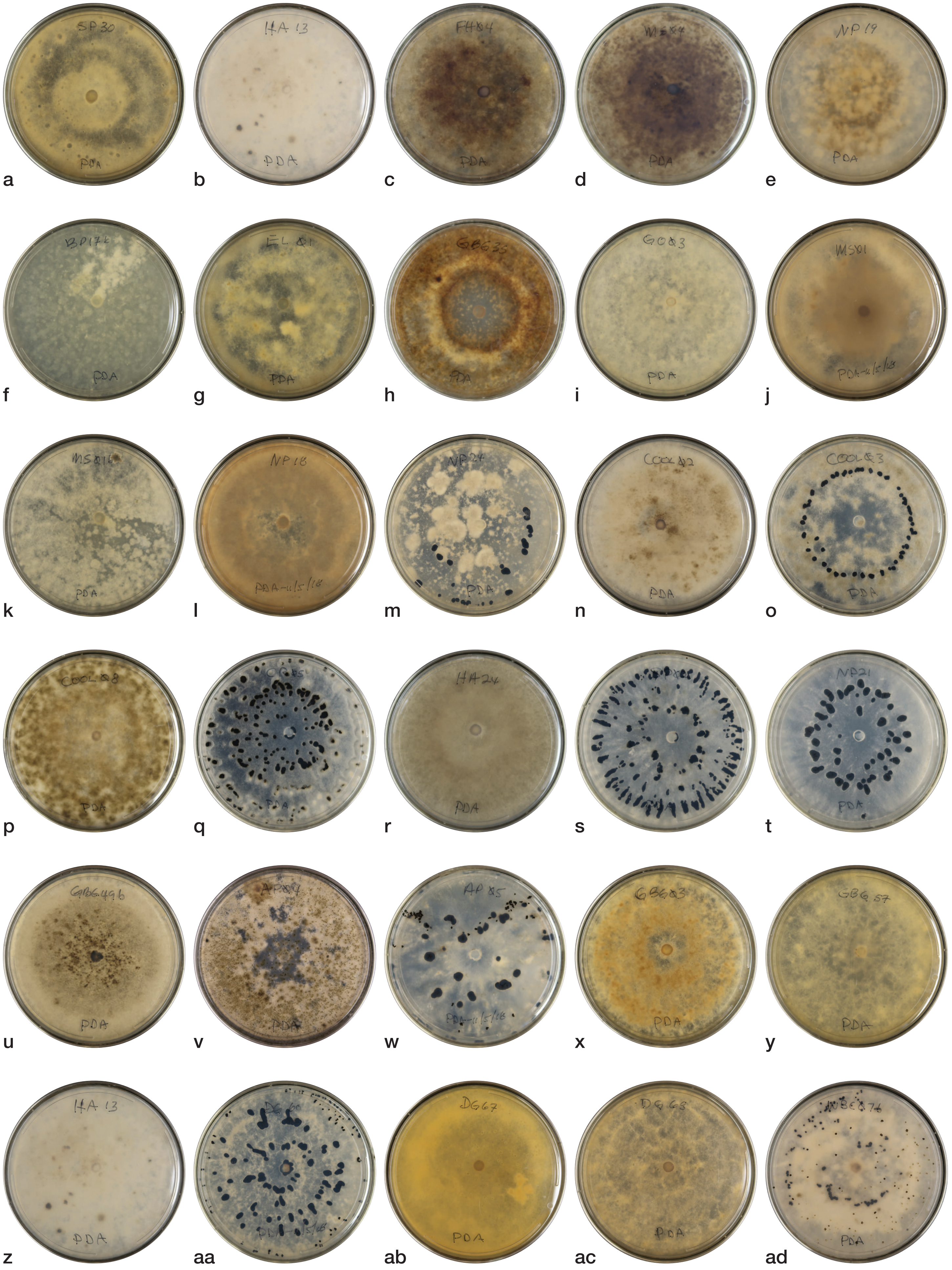
a
b
c
d
e
f
g
h
i
j
k
l
m
n
o
p
q
r
s
t
u
v
w
x
y
z
aa
ab
ac
ad

Supplementary Fig. 7 *Botrytis* spp. isolates (a) SP30, (b) HA13, (c) FH02, (d) MS04, (e) NP19, (f) BP17b, (g) EL01, (h) GBG35, (i) GO03, (j) MS01, (k), MS01b, (l) NP18, (m) NP24, (n), COOL02, (o) COOL03, (p) COOL08, (q) CR05, (r) HA24, (s) SP02, (t) NP21, (u) GBG49b, (v) AP04, (w) AP05, (x) GBG02, (y) GBG57, (z) DG37, (aa) DG60, (ab) DG67, (ac) DG68, and (ad) WBC07c collected from peonies grown on potato dextrose agar for 21 days in the dark at 20 C. Photographs are of the bottom of the culture plate.

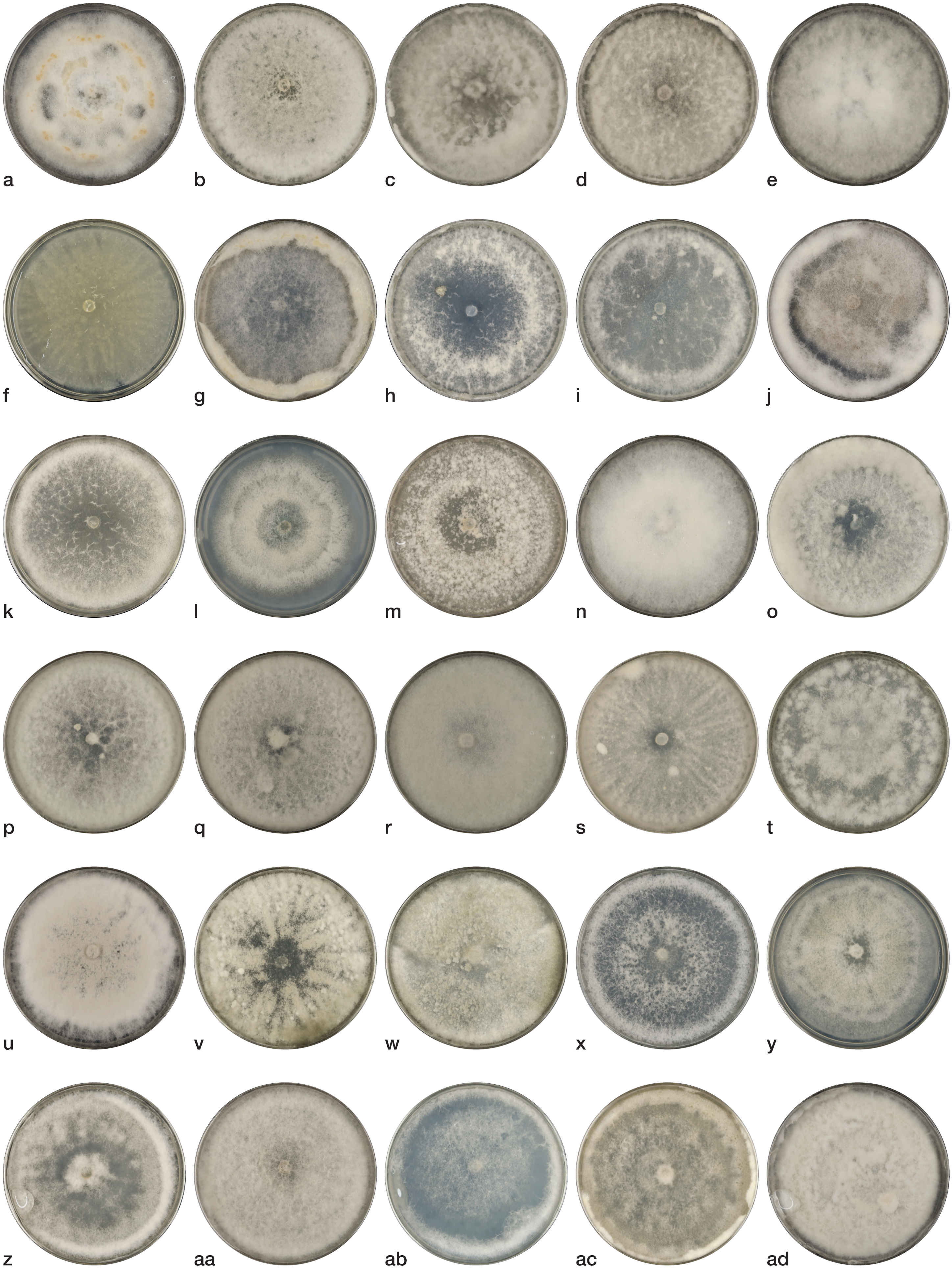

Supplementary Fig. 8 *Botrytis* spp. isolates (a) SP30, (b) HA13, (c) FH02, (d) MS04, (e) NP19, (f) BP17b, (g) EL01, (h) GBG35, (i) GO03, (j) MS01, (k), MS01b, (l) NP18, (m) NP24, (n), COOL02, (o) COOL03, (p) COOL08, (q) CR05, (r) HA24, (s) SP02, (t) NP21, (u) GBG49b, (v) AP04, (w) AP05, (x) GBG02, (y) GBG57, (z) DG37, (aa) DG60, (ab) DG67, (ac) DG68, and (ad) WBC07c collected from peonies grown on potato dextrose agar for 7 days under constant ultra-violet (UV) light at 20 C.

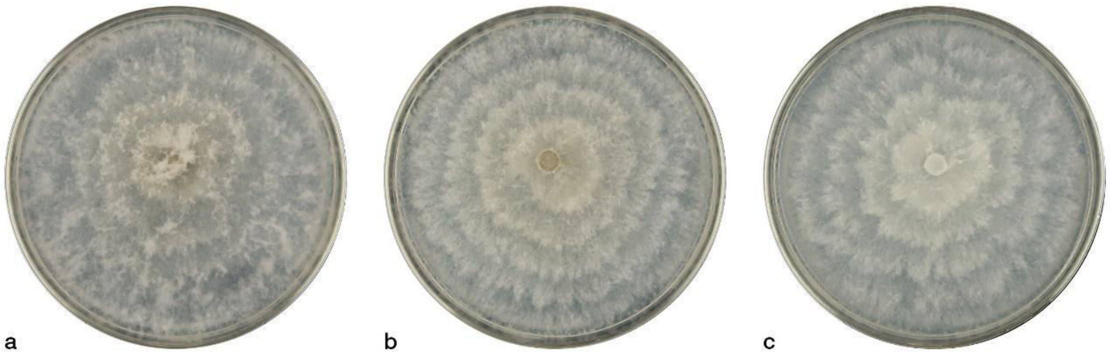

Supplementary Fig. S9 Seven-day-old cultures of isolates (a) FH04, (b) MS04, and (c) NP19 grown on malt extract agar at 20 C in the dark. All isolates are representatives of the same phylogenetic cluster and show a characteristic stellate growth pattern not evident under any other conditions tested or in other isolates collected in this study.

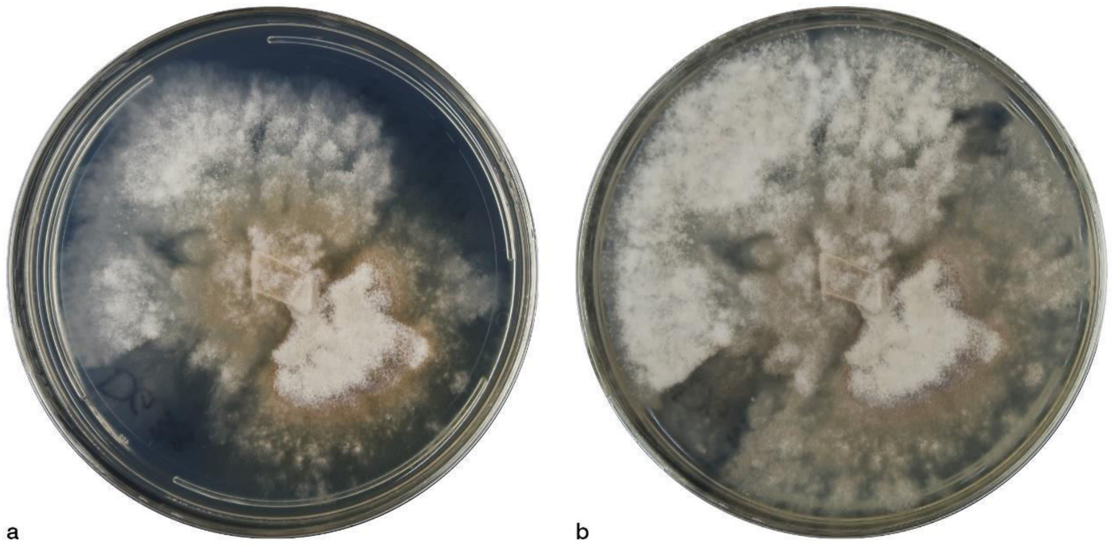

Supplementary Fig. S10 Isolate DG38 from peony at (a) 7 and (b) 14 days grown on potato dextrose agar at 20 C in the dark.

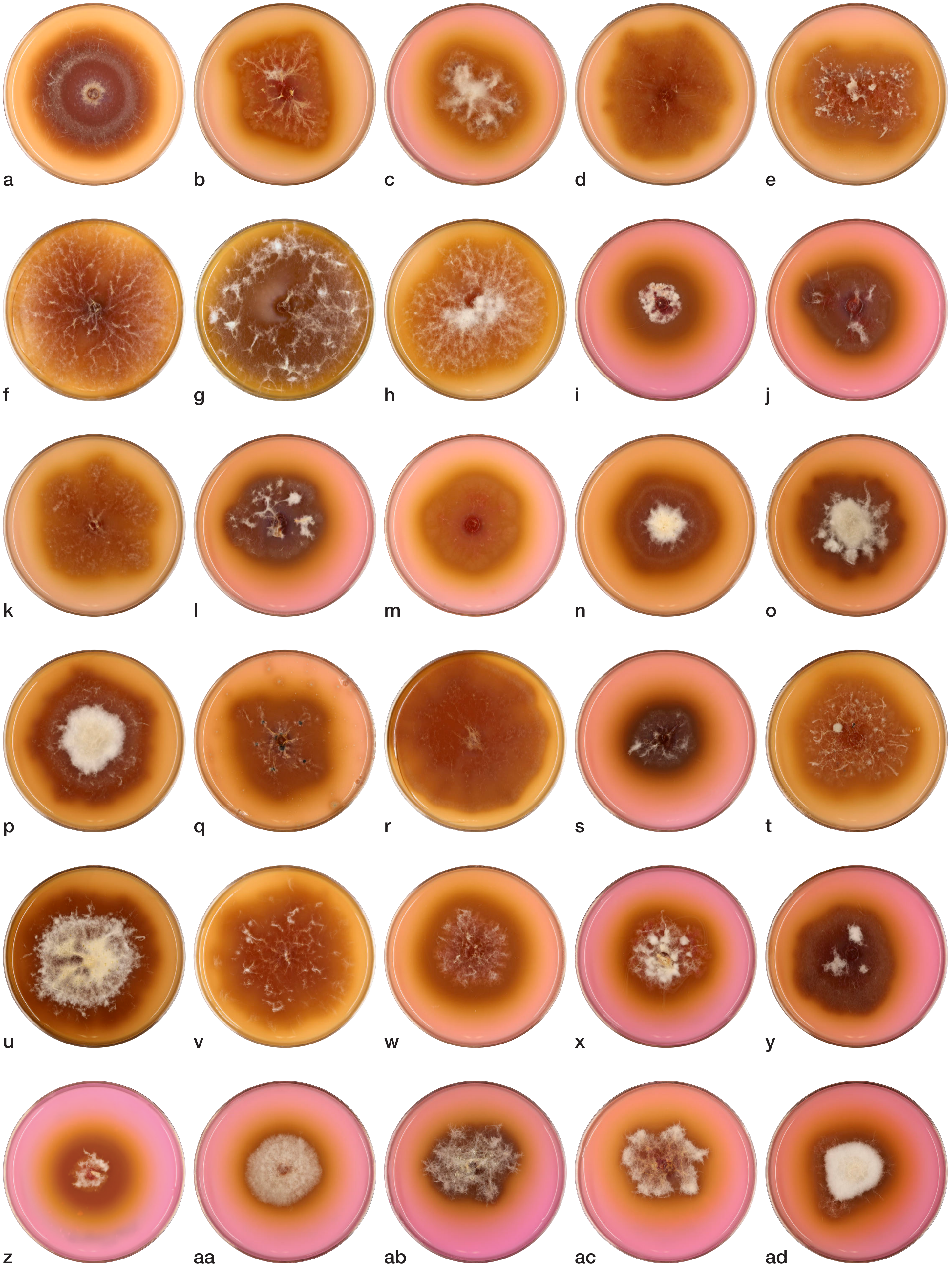
a
b
c
d
e
f
g
h
i
j
k
l
m
n
o
p
q
r
s
t
u
v
w
x
y
z
aa
ab
ac
ad

Supplementary Fig. 11 *Botrytis* spp. isolates (a) SP30, (b) HA13, (c) FH02, (d) MS04, (e) NP19, (f) BP17b, (g) EL01, (h) GBG35, (i) GO03, (j) MS01, (k), MS01b, (l) NP18, (m) NP24, (n), COOL02, (o) COOL03, (p) COOL08, (q) CR05, (r) HA24, (s) SP02, (t) NP21, (u) GBG49b, (v) AP04, (w) AP05, (x) GBG02, (y) GBG57, (z) DG37, (aa) DG60, (ab) DG67, (ac) DG68, and (ad) WBC07c collected from peonies grown on a *Botrytis*-specific medium for 21 days in the dark at 20 C.

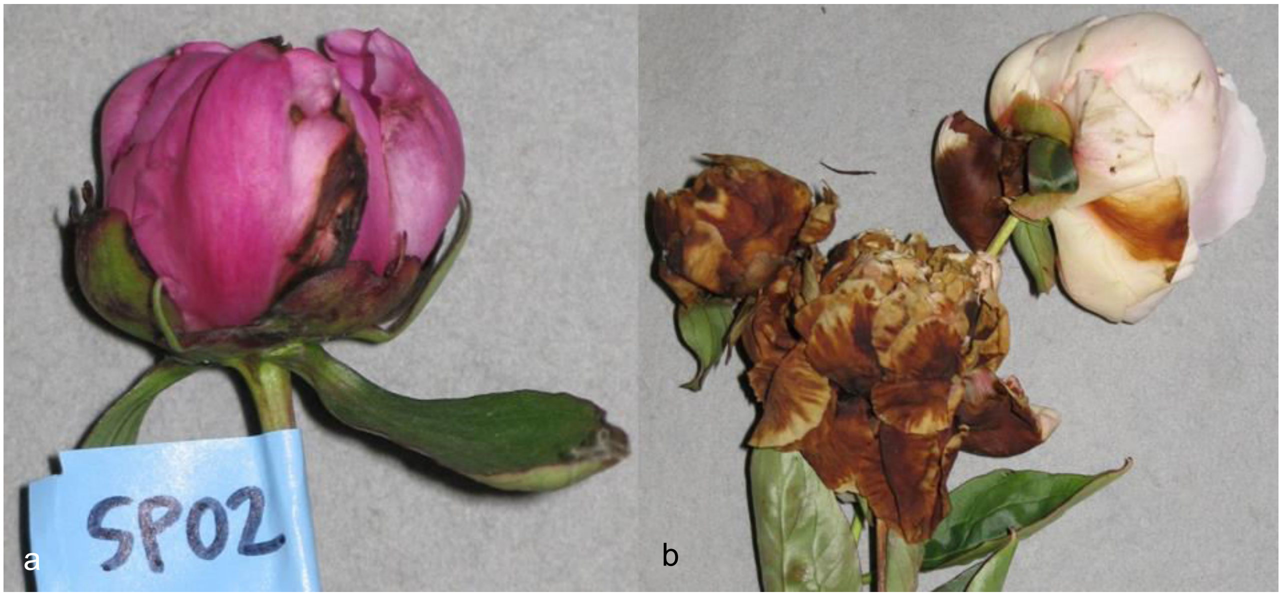

Supplementary Fig. S12 Peony samples from which *Botrytis* isolates (a) SP02 and (b) NF13 were isolated showing symptoms of flower decay involving a brown rot of petal tissue.

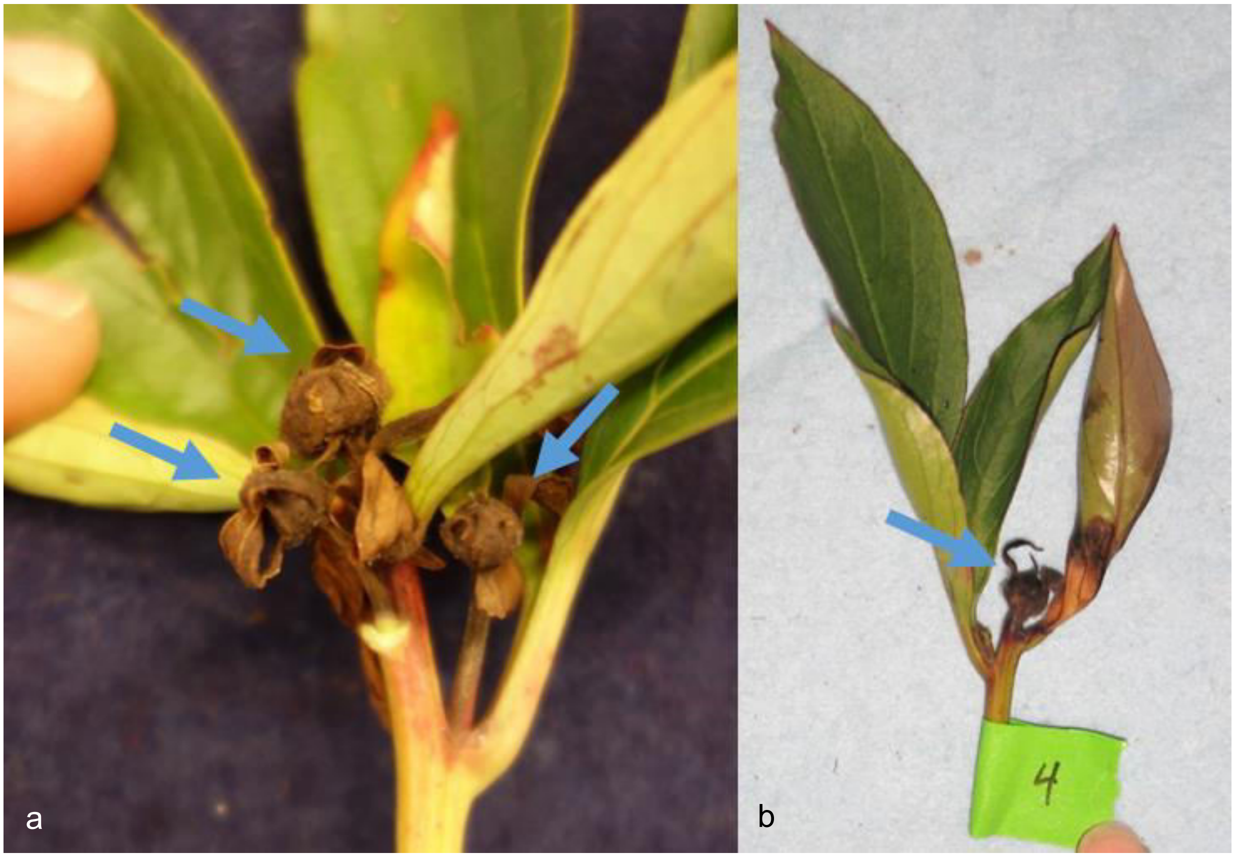

Supplementary Fig. S13 Peony samples from which *Botrytis* isolates (a) DG67 and (b) NP19 were isolated showing blasted buds (indicated by blue arrows) which are small, under-developed flower buds. It is possible the buds died due to physiological reasons prior to colonization by *Botrytis*.

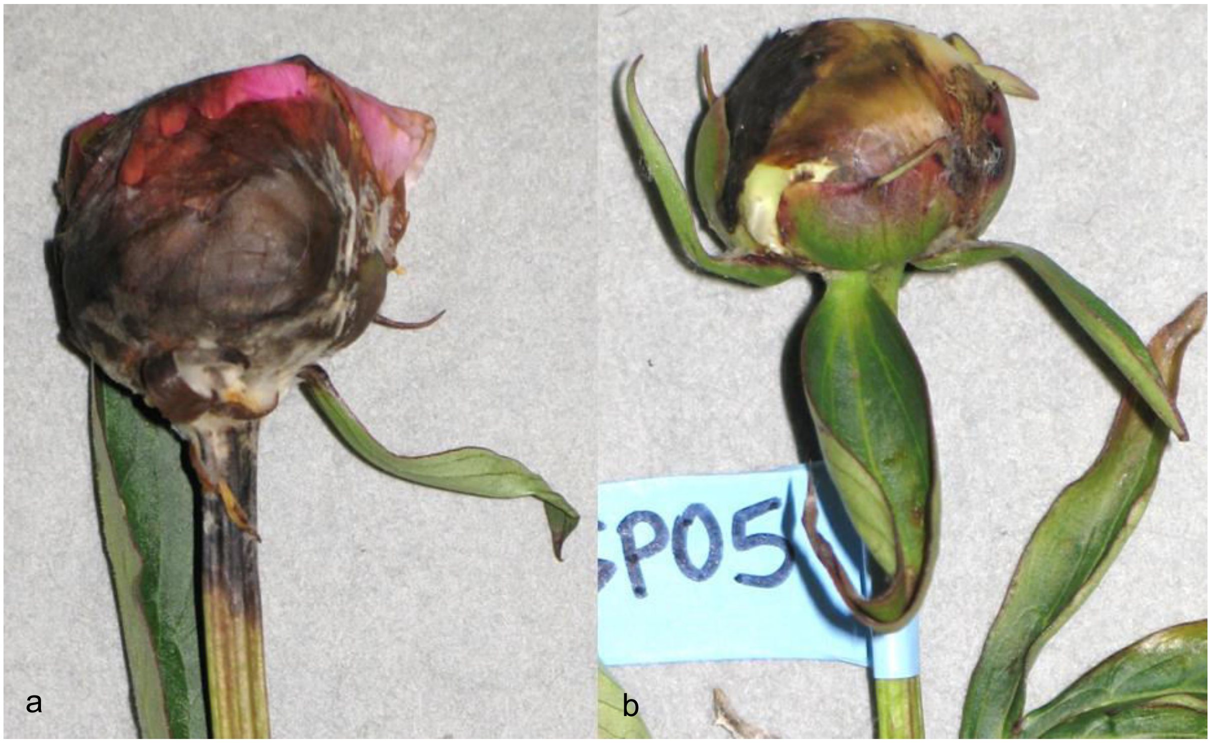

Supplementary Fig. S14 Peony samples from which *Botrytis* isolates (a) MS01 and (b) SP05 were isolated showing flower bud decay in which the sepals have been decayed on a fully closed or partially-opened flower bud, but prior to petal expansion.

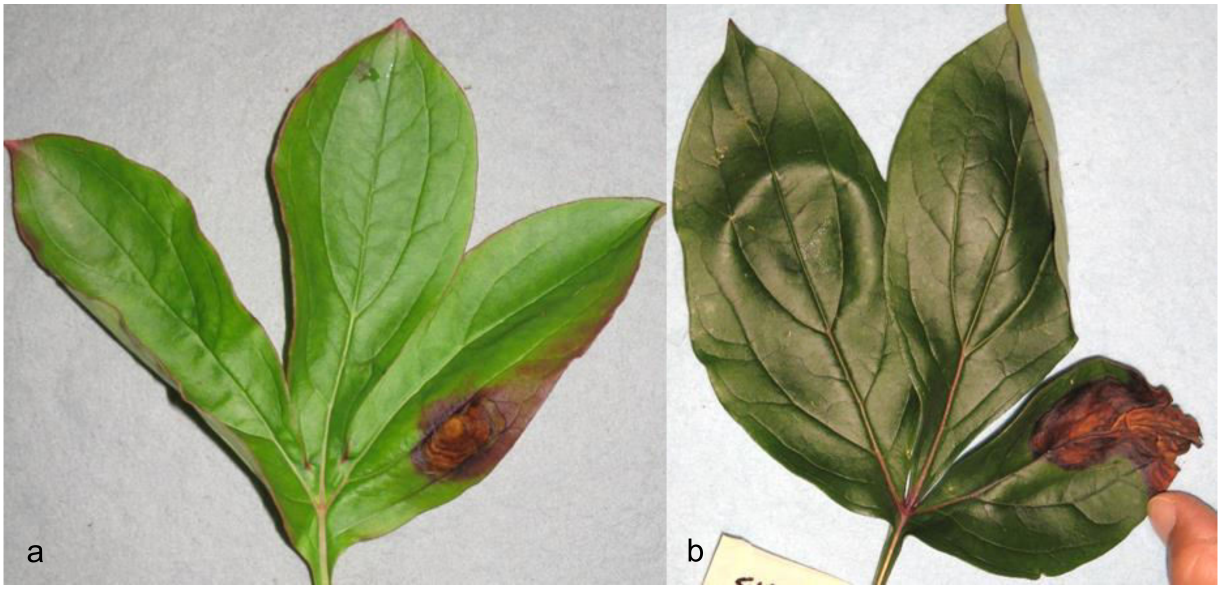

Supplementary Fig. S15 Peony samples from which *Botrytis* isolates (a) COOL08 and (b) SH06 were isolated with a tan-brown to dark brown foliar lesions. Both samples show distinct banding of alternating dark and light brown within the lesion. While not always evident, these so-called "zonate" lesions are characteristic of *Botrytis* lesions on peony on both leaf and stem tissue.

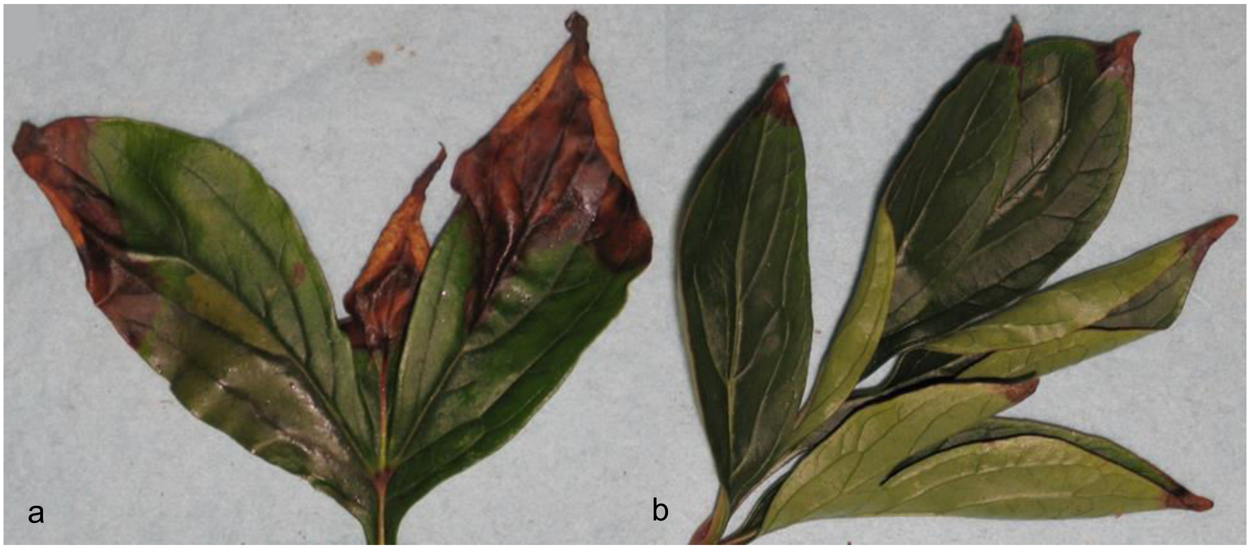

Supplementary Fig. S16 Peony samples from which *Botrytis* isolates (a) NP21 and (b) FH04 were isolated showing symptoms of leaf tip dieback characterized by a necrosis originating at the tips of the leaflets.

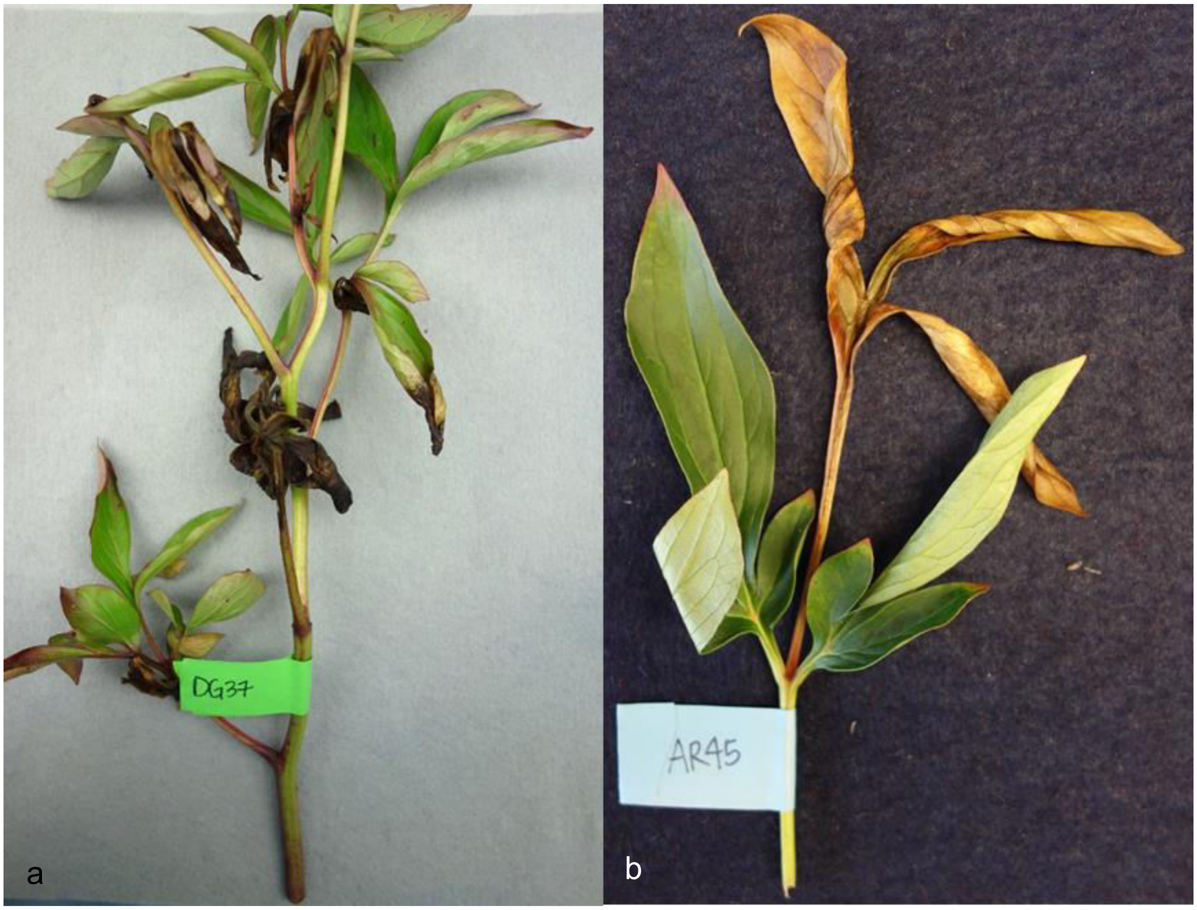

Supplementary Fig. S17 Peony samples from which *Botrytis* isolates (a) DG37 and (b) AR45 were isolated showing symptoms of foliar dieback in which leaves have become completely necrotic (no distinct lesion is evident) and in which the necrosis has started to move into the petiole, but has not yet reached the stem.

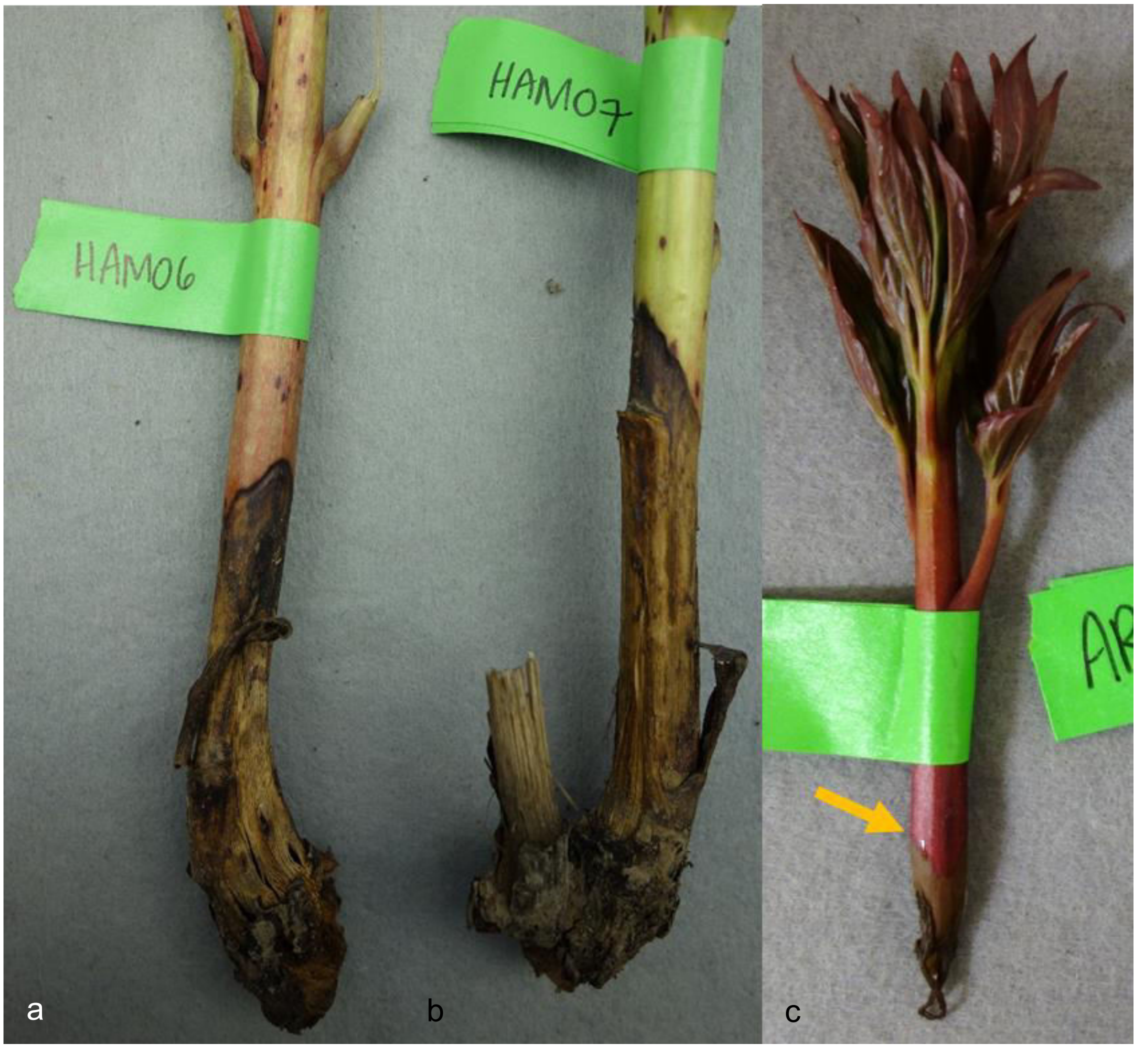

Supplementary Fig. S18 Peony samples from which *Botrytis* isolates (a) HAM06 and (b) HAM07 (sample not included in study) were isolated showing symptoms of basal stem decay where the base of the shoot has a lesion caused by *Botrytis*. The infection can be seen at and below the soil level and moves up the stem towards the flowering end of the shoot. In (b), parts of the root crown, including the previous years' shoots are still connected at the base of the new shoot. (c) Basal stem decay can also be seen on newly emerging shoots such as in sample AR20 (lesion margin indicated by yellow arrow).

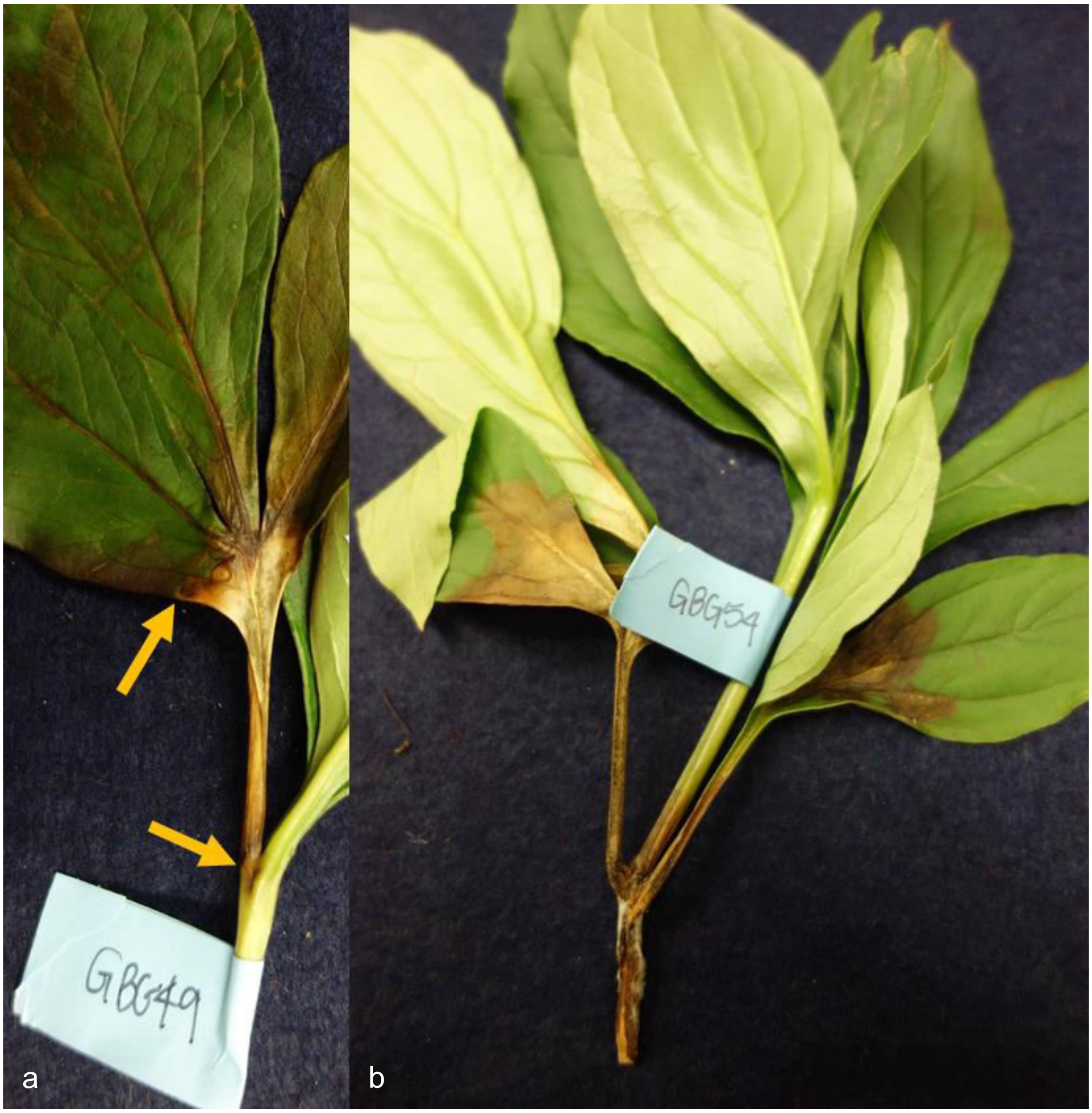

Supplementary Fig. S19 Peony samples from which *Botrytis* isolates (a) GBG49b and (b) GBG54 were isolated with lesions at the petiole in which infection appears to have originated at or close to the petiole and the fungus has subsequently colonized the petiole and leaf tissue. (a) On sample GBG49b, the margins of the lesion are indicated by the two yellow arrows.

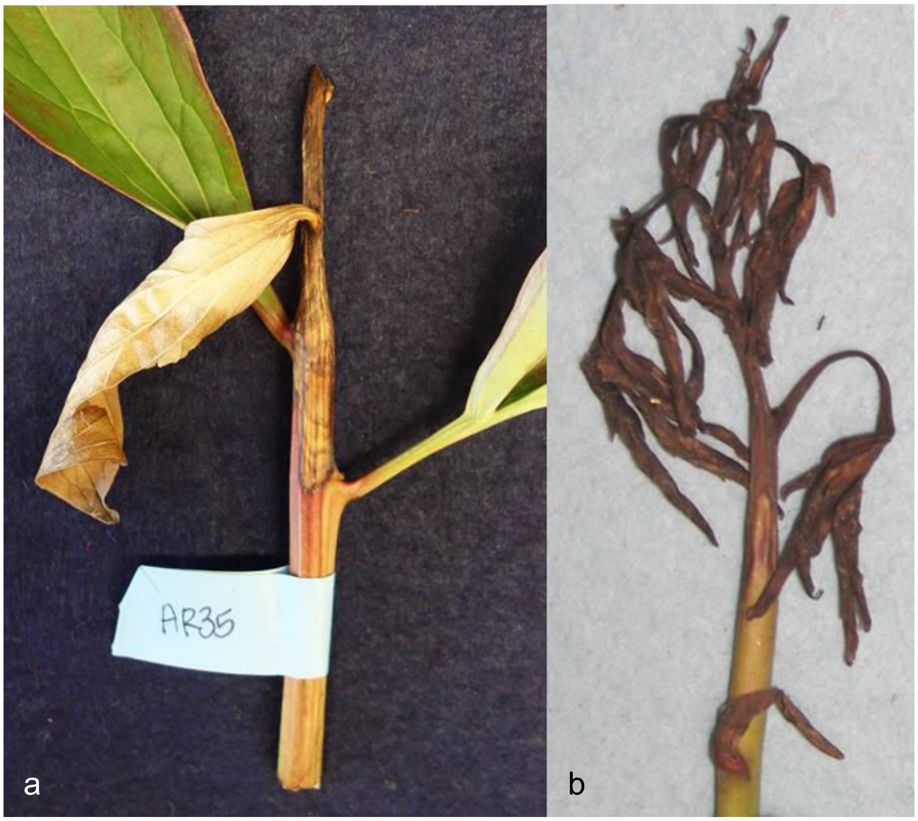

Supplementary Fig. S20 Peony samples from which *Botrytis* isolates (a) AR35 and (b) BP17b were isolated showing symptoms of shoot dieback. Original infection has likely started in the foliage or bud and has moved down into the stem tissue.

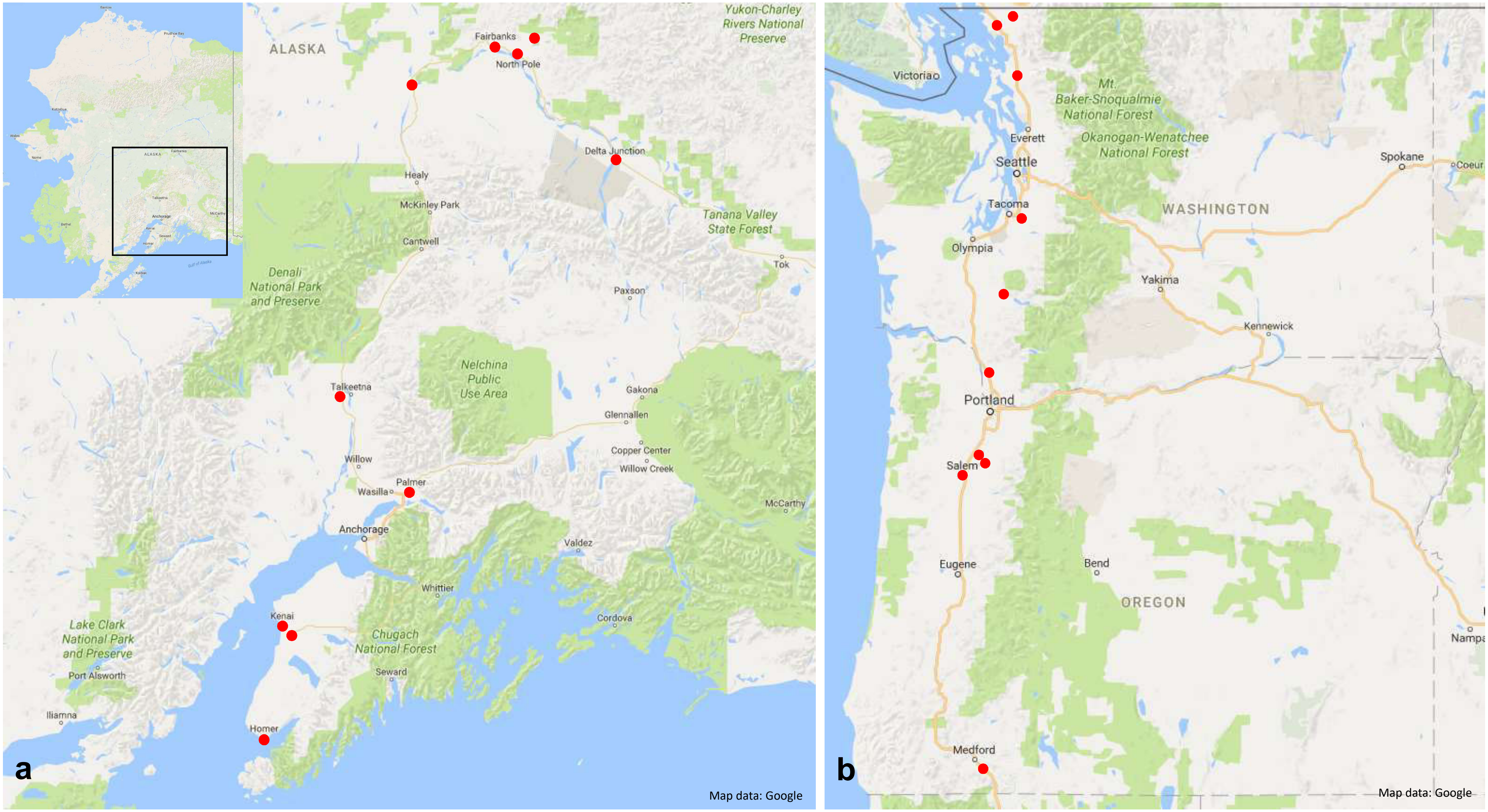
ALASKA
Fairbanks
North Pole
Yukon-Charley Rivers National Preserve
Delta Junction
Healy
McKinley Park
Cantwell
Tanana Valley State Forest
Tok
Denali National Park and Preserve
Paxson
Nelchina Public Use Area
Talkeetna
Gakona
Glennallen
Copper Center
Willow Creek
Willow
Palmer
Wasilla
McCarthy
Anchorage
Valdez
Whittier
Kenai
Cordova
Chugach National Forest
Seward
Lake Clark National Park and Preserve
Port Alsworth
Iliamna
Homer
a
Map data: Google
Victoria
Mt. Baker-Snoqualmie National Forest
Everett
Okanogan-Wenatchee National Forest
Seattle
Spokane
Coeur
Tacoma
WASHINGTON
Olympia
Yakima
Kennewick
Portland
Salem
Eugene
Bend
OREGON
Nampa
Medford
b
Map data: Google

Supplementary Fig. 21 Approximate survey locations (indicated by red dot) in (a) Alaska and (b) Washington and Oregon. In some instances, multiple farms were surveyed in a single survey location.

Supplementary Table S1 List of *Botrytis* isolates collected from peony during the 2014 and 2015 field season in Washington, Oregon, and Alaska. GenBank accession numbers are provided for isolates used in phylogenetic analysis.

Isolate	Species	Collection Location	Symptom	Collection Year	G3PDH	$HSP60_a$	$RPB2_a$	$NEP1_a$	$NEP2_a$
AB01	*B. paeoniae*	Alaska	Basal stem decay	2014	KY200340	–	–	–	–
AB03	*B. pseudocinerea*	Alaska	Blasted bud	2014	KY200341	MK736283	–	–	–
AD07	*B. cinerea*	Oregon	Blasted bud	2014	KY200342	–	–	–	–
AD12	*B. cinerea*	Oregon	Leaf tip necrosis	2014	KY200343	–	–	–	–
AP04	AKBot8	Alaska	Blasted bud	2014	KY200344	KY203864	KY211622	KY553167	KY230655
AP05	AKBot8	Alaska	Foliar lesion	2014	KY200345	KY203865	KY211623	KY553168	KY230656
AP06	*B. cinerea*	Alaska	Leaf tip necrosis	2014	KY200346	–	–	–	–
AR05	*B. paeoniae*	Washington	Basal stem decay	2014	KY200347	–	–	–	–
AR06	*B. paeoniae*	Washington	Basal stem decay	2014	KY200348	–	–	–	–
AR08	*B. paeoniae*	Washington	Shoot dieback	2014	KY200349	–	–	–	–
AR11	*B. cinerea*	Washington	Blasted bud	2014	KY200350	–	–	–	–
AR20	*B. paeoniae*	Washington	Basal stem decay	2015	KY200351	–	–	–	–
AR24	*B. paeoniae*	Washington	Basal stem decay	2015	KY200352	–	–	–	–
AR35	*B. paeoniae*	Washington	Shoot dieback	2015	KY200353	–	–	–	–
AR36	*B. paeoniae*	Washington	Foliar dieback	2015	KY200354	–	–	–	–
AR38	*B. cinerea*	Washington	Foliar dieback	2015	KY200355	–	–	–	–
AR45	*B. paeoniae*	Washington	Foliar dieback	2015	KY200356	–	–	–	–
AR46	*B. cinerea*	Washington	Blasted bud	2015	KY200357	–	–	–	–
AR47	*B. cinerea*	Washington	Shoot dieback	2015	KY200358	–	–	–	–
AR48	*B. paeoniae*	Washington	Shoot dieback	2015	KY200359	–	–	–	–
AR49	*B. paeoniae*	Washington	Shoot dieback	2015	KY200360	–	–	–	–
AR50	*B. paeoniae*	Washington	Flower decay	2015	KY200361	–	–	–	–
BF08	*B. cinerea*	Alaska	Blasted bud	2014	KY200362	–	–	–	–
BP17b	AKBot4	Alaska	Shoot dieback	2014	KY200363	KY203866	KY211624	KY553169	KY230657
BP18	*B. euroamericana*	Alaska	Blasted bud	2014	KY200364	KY203867	KY211625	KY553170	KY230658
BP18b	*B. euroamericana*	Alaska	Foliar decay	2014	KY200365	KY203868	KY211626	KY553171	KY230659
BP21	*B. euroamericana*	Alaska	Foliar lesion	2014	KY200366	KY203869	KY211627	KY553172	KY230660
BP22	*B. euroamericana*	Alaska	Foliar lesion	2014	KY200367	KY203870	KY211628	KY553173	KY230661
BP30	*B. cinerea*	Alaska	Foliar lesion	2015	KY200368	–	–	–	–
CC03	*B. cinerea*	Alaska	Foliar lesion	2014	KY200369	–	–	–	–

COOL02	AKBot5	Alaska	Foliar lesion	2014	KY200370	KY203871	KY211629	KY553174	KY230662
COOL03	AKBot5	Alaska	Foliar lesion	2014	KY200371	KY203872	KY211630	KY553175	KY230663
COOL08	AKBot5	Alaska	Foliar lesion	2014	KY200372	KY203873	KY211631	KY553176	KY230664
COOL09	*B. paeoniae*	Alaska	Shoot dieback	2014	KY200373	–	–	–	–
CR02	*B. cinerea*	Alaska	Blasted bud	2014	KY200374	–	–	–	–
CR05	AKBot5	Alaska	Foliar lesion	2014	KY200375	KY203874	KY211632	KY553177	KY230665
CR06	*B. cinerea*	Alaska	Foliar lesion	2014	KY200376	–	–	–	–
DG05	*B. cinerea*	Washington	Flower bud decay	2014	KY200377	–	–	–	–
DG06	*B. paeoniae*	Washington	Basal stem decay	2014	KY200378	–	–	–	–
DG08	*B. paeoniae*	Washington	Foliar dieback	2014	KY200379	–	–	–	–
DG10	*B. paeoniae*	Washington	Flower bud decay	2014	KY200380	–	–	–	–
DG13	*B. cinerea*	Washington	Flower bud decay	2014	KY200381	–	–	–	–
DG24	*B. paeoniae*	Washington	Basal stem decay	2014	KY200382	–	–	–	–
DG29	*B. paeoniae*	Washington	Foliar lesion	2015	KY200383	–	–	–	–
DG30	*B. paeoniae*	Washington	Shoot tip dieback	2015	KY200384	–	–	–	–
DG37	WABot1	Washington	Foliar dieback	2015	KY200385	KY203875	KY211633	KY553178	KY230666
DG38	WABot3	Washington	Foliar lesion	2015	KY200386	KY203876	KY211634	KY553179	KY230667
DG40	*B. pseudocinerea*	Washington	Foliar lesion	2015	KY200387	MK736284	–	–	–
DG42	*B. paeoniae*	Washington	Flower bud decay	2015	KY200388	–	–	–	–
DG46	*B. paeoniae*	Washington	Foliar dieback	2015	KY200389	–	–	–	–
DG47	*B. paeoniae*	Washington	Flower bud decay	2015	KY200390	–	–	–	–
DG60	WABot2	Washington	Foliar dieback	2015	KY200391	KY203877	KY211635	KY553180	KY230668
DG64	*B. paeoniae*	Washington	Flower decay	2015	KY200392	–	–	–	–
DG67	WABot2	Washington	Blasted bud	2015	KY200393	KY203878	KY211636	KY553181	KY230669
DG68	WABot2	Washington	Blasted bud	2015	KY200394	KY203879	KY211637	KY553182	KY230670
DL07	*B. cinerea*	Alaska	Foliar lesion	2014	KY200395	–	–	–	–
EL01	AKBot4	Alaska	Foliar lesion	2014	KY200396	KY203880	KY211638	KY553183	KY230671
EL03	*B. cinerea*	Alaska	Leaf tip dieback	2014	KY200397	–	–	–	–
EL05	*B. cinerea*	Alaska	Flower bud decay	2014	KY200398	–	–	–	–
FH01	*B. cinerea*	Alaska	Leaf tip tieback	2014	KY200399	–	–	–	–
FH04	AKBot3	Alaska	Leaf tip dieback	2014	KY200400	KY203881	KY211639	KY553184	KY230672
GBG01	*B. cinerea*	Alaska	Flower decay	2014	KY200401	–	–	–	–
GBG03	AKBot9	Alaska	Basal stem decay	2014	KY200402	KY203882	KY211640	KY553185	KY230673
GBG05	*B. paeoniae*	Alaska	Shoot dieback	2014	KY200403	–	–	–	–
GBG09	*B. cinerea*	Alaska	Foliar lesion	2014	KY200404	–	–	–	–

GBG10	*B. cinerea*	Alaska	Foliar lesion	2014	KY200405	–	–	–	–
GBG22	*B. paeoniae*	Alaska	Basal stem decay	2014	KX266730	–	–	–	–
GBG31b	*B. paeoniae*	Alaska	Foliar lesion	2015	KY200406	–	–	–	–
GBG32	*B. paeoniae*	Alaska	Foliar lesion	2015	KY200407	–	–	–	–
GBG35	AKBot4	Alaska	Foliar lesion	2015	KY200408	KY203883	KY211641	KY553186	KY230674
GBG40	*B. paeoniae*	Alaska	Foliar lesion	2015	KY200409	–	–	–	–
GBG41	*B. paeoniae*	Alaska	Foliar lesion	2015	KY200410	–	–	–	–
GBG46	*B. paeoniae*	Alaska	Foliar lesion	2015	KY200411	–	–	–	–
GBG49b	AKBot7	Alaska	Lesion at petiole	2015	KY200412	KY203884	KY211642	KY553187	KY230675
GBG50	*B. paeoniae*	Alaska	Foliar lesion	2015	KY200413	–	–	–	–
GBG52	*B. paeoniae*	Alaska	Foliar lesion	2015	KY200414	–	–	–	–
GBG54	*B. paeoniae*	Alaska	Lesion at petiole	2015	KY200415	–	–	–	–
GBG55	*B. paeoniae*	Alaska	Lesion at petiole	2015	KY200416	–	–	–	–
GBG57	AKBot9	Alaska	Shoot dieback	2015	KY200417	KY203885	KY211643	KY553188	KY230676
GBG61	*B. cinerea*	Alaska	Shoot dieback	2015	KY200418	–	–	–	–
GO01	*B. cinerea*	Alaska	Flower decay	2014	KY200419	–	–	–	–
GO02	*B. euroamericana*	Alaska	Flower bud decay	2014	KY200420	KY203886	KY211644	KY553189	KY230677
GO03	AKBot4	Alaska	Flower bud decay	2014	KY200421	KY203887	KY211645	KY553190	KY230678
GO04	*B. cinerea*	Alaska	Foliar lesion	2014	KY200422	–	–	–	–
HA05	*B. paeoniae*	Alaska	Basal stem decay	2014	KY200423	–	–	–	–
HA08	*B. cinerea*	Alaska	Foliar lesion	2014	KX266731	–	–	–	–
HA11	*B. paeoniae*	Alaska	Foliar lesion	2014	KX266729	–	–	–	–
HA13	AKBot2	Alaska	Flower bud decay	2014	KY200424	KY203888	KY211646	KY553191	KY230679
HA14	*B. cinerea*	Alaska	Flower bud decay	2014	KY200425	–	–	–	–
HA18	*B. euroamericana*	Alaska	Leaf tip dieback	2015	KY200426	KY203889	KY211647	KY553192	KY230680
HA24	AKBot5	Alaska	Shoot dieback	2015	KY200427	KY203890	KY211648	KY553193	KY230681
HA29b	*B. euroamericana*	Alaska	Foliar dieback	2015	KY200428	KY203891	KY211649	KY553194	KY230682
HAM01	*B. paeoniae*	Oregon	Shoot dieback	2015	KY200429	–	–	–	–
HAM06	*B. paeoniae*	Oregon	Basal stem decay	2015	KY200430	–	–	–	–
HAM08	*B. pseudocinerea*	Oregon	Foliar dieback	2015	KY200431	MK736285	–	–	–
HF05	*B. cinerea*	Alaska	Foliar lesion	2014	KY200432	–	–	–	–
HF07	*B. cinerea*	Alaska	Foliar lesion	2014	KY200433	–	–	–	–
JM01A	*B. paeoniae*	Washington	Basal stem decay	2014	KY200434	–	–	–	–
JM02	*B. paeoniae*	Washington	Basal stem decay	2014	KY200435	–	–	–	–
JM02b	*B. pseudocinerea*	Washington	Flower bud decay	2014	KY200436	MK736286	–	–	–

JM03	*B. cinerea*	Washington	Shoot dieback	2014	KY200437	–	–	–	–
KC08b	*B. paeoniae*	Washington	Foliar dieback	2015	KY200438	–	–	–	–
LM05	*B. paeoniae*	Oregon	Blasted bud	2015	KY200439	–	–	–	–
LM05b	*B. paeoniae*	Oregon	Leaf tip dieback	2015	KY200440	–	–	–	–
LP01	*B. paeoniae*	Alaska	Foliar lesion	2015	KY200441	–	–	–	–
LP03	*B. paeoniae*	Alaska	Foliar dieback	2015	KY200442	–	–	–	–
MS01	AKBot4	Alaska	Flower bud decay	2014	KY751717	KY751719	KY751721	KY553195	KY751723
MS01b	AKBot4	Alaska	Shoot dieback	2014	KY200443	KY203892	KY211650	KY553196	KY230683
MS04	AKBot3	Alaska	Blasted bud	2014	KY200444	KY203893	KY211651	KY553197	KY230684
MS05	*B. cinerea*	Alaska	Flower bud decay	2014	KX266732	–	–	–	–
MS06	*B. cinerea*	Alaska	Flower decay	2014	KY200445	–	–	–	–
MS09	*B. paeoniae*	Alaska	Foliar lesion	2014	KY200446	–	–	–	–
MS10	*B. cinerea*	Alaska	Foliar lesion	2014	KY200447	–	–	–	–
MS11	*B. cinerea*	Alaska	Foliar lesion	2014	KY200448	–	–	–	–
MS12	*B. cinerea*	Alaska	Flower decay	2014	KY200449	–	–	–	–
MV01	*B. pseudocinerea*	Washington	Foliar lesion	2014	KY200450	MK736287	–	–	–
MV02	*B. cinerea*	Washington	Foliar lesion	2014	KY200451	–	–	–	–
MV03	*B. pseudocinerea*	Washington	Foliar lesion	2014	KY200452	MK736288	–	–	–
MV04	*B. cinerea*	Washington	Foliar lesion	2014	KY200453	–	–	–	–
MV05	*B. pseudocinerea*	Washington	Foliar lesion	2014	KY200454	MK736289	–	–	–
NF08	*B. cinerea*	Washington	Foliar lesion	2014	KY200455	–	–	–	–
NF13	*B. cinerea*	Washington	Flower decay	2014	KY200456	–	–	–	–
NF16	*B. cinerea*	Washington	Foliar dieback	2014	KY200457	–	–	–	–
NF18	*B. cinerea*	Washington	Foliar dieback	2014	KY200458	–	–	–	–
NP16	*B. euroamericana*	Alaska	Blasted bud	2014	KY200459	KY203894	KY211652	KY553198	KY230685
NP18	AKBot4	Alaska	Blasted bud	2014	KY751718	KY751720	KY751722	KY553199	KY751724
NP19	AKBot3	Alaska	Blasted bud	2014	KY200460	KY203895	KY211653	KY553200	KY230686
NP21	AKBot6	Alaska	Leaf tip dieback	2014	KY200461	KY203896	KY211654	KY553201	KY230687
NP22	*B. cinerea*	Alaska	Foliar lesion	2014	KY200462	–	–	–	–
NP24	AKBot4	Alaska	Foliar lesion	2014	KY200463	KY203897	KY211655	KY553202	KY230688
OP01	*B. pseudocinerea*	Oregon	Foliar lesion	2014	KY200464	MK736290	–	–	–
OP07	*B. cinerea*	Oregon	Foliar dieback	2014	KY200465	–	–	–	–
OP14	*B. paeoniae*	Oregon	Basal stem decay	2014	KY200466	–	–	–	–
OP15	*B. cinerea*	Oregon	Flower decay	2014	KY200467	–	–	–	–
OP27	*B. pseudocinerea*	Oregon	Foliar dieback	2015	KY200468	MK736291	–	–	–

OP28b	*B. pseudocinerea*	Oregon	Foliar dieback	2015	KY200469	MK736292	–	–	–
OP46	*B. paeoniae*	Oregon	Lesion at petiole	2015	KY200470	–	–	–	–
OP47	*B. paeoniae*	Oregon	Bud dieback	2015	KY200471	–	–	–	–
OP50	*B. paeoniae*	Oregon	Bud dieback	2015	KY200472	–	–	–	–
OP52	*B. pseudocinerea*	Oregon	Foliar lesion	2015	KY200473	MK736293	–	–	–
OP54	*B. cinerea*	Oregon	Foliar lesion	2015	KY200474	–	–	–	–
OP57	*B. paeoniae*	Oregon	Shoot dieback	2015	KY200475	–	–	–	–
OP60	*B. cinerea*	Oregon	Lesion at petiole	2015	KY200476	–	–	–	–
OP61	*B. cinerea*	Oregon	Blasted bud	2015	KY200477	–	–	–	–
PE01	*B. cinerea*	Alaska	Blasted bud	2014	KY200478	–	–	–	–
PIO01	*B. cinerea*	Alaska	Foliar lesion	2014	KY200479	–	–	–	–
PP08	*B. cinerea*	Alaska	Flower bud decay	2014	KY200480	–	–	–	–
PP11	*B. cinerea*	Alaska	Flower bud decay	2014	KY200481	–	–	–	–
PP15	*B. cinerea*	Alaska	Foliar lesion	2014	KY200482	–	–	–	–
PUY09	*B. cinerea*	Washington	Foliar lesion	2015	KY200483	–	–	–	–
PUY17	*B. paeoniae*	Washington	Basal stem decay	2015	KY200484	–	–	–	–
PUY18	*B. paeoniae*	Washington	Basal stem decay	2015	KY200485	–	–	–	–
PUY20	*B. paeoniae*	Washington	Blasted bud	2015	KY200486	–	–	–	–
SH01	*B. euroamericana*	Alaska	Flower bud decay	2014	KY200487	KY203898	KY211656	KY553203	KY230689
SH02	*B. euroamericana*	Alaska	Flower bud decay	2014	KY200488	KY203899	KY211657	KY553204	KY230690
SH03	*B. cinerea*	Alaska	Blasted bud	2014	KY200489	–	–	–	–
SH06	*B. euroamericana*	Alaska	Foliar lesion	2014	KY200490	KY203900	KY211658	KY553205	KY230691
SP02	AKBot5	Alaska	Flower decay	2014	KY200491	KY203901	KY211659	KY553206	KY230692
SP03	*B. cinerea*	Alaska	Flower decay	2014	KY200492	–	–	–	–
SP05	*B. cinerea*	Alaska	Flower bud decay	2014	KY200493	–	–	–	–
SP05b	*B. cinerea*	Alaska	Leaf tip dieback	2014	KY200494	–	–	–	–
SP27	*B. cinerea*	Alaska	Foliar lesion	2014	KY200495	–	–	–	–
SP28	*B. cinerea*	Alaska	Foliar lesion	2014	KY200496	–	–	–	–
SP30	AKBot1	Alaska	Blasted bud	2014	KY200497	KY203902	KY211660	KY553207	KY230693
SP32	*B. cinerea*	Alaska	Foliar lesion	2014	KY200498	–	–	–	–
SP33	*B. paeoniae*	Alaska	Flower decay	2014	KY200499	–	–	–	–
SP34	*B. paeoniae*	Alaska	Basal stem decay	2015	KY200500	–	–	–	–
SP36	*B. paeoniae*	Alaska	Basal stem decay	2015	KY200501	–	–	–	–
SP37	*B. paeoniae*	Alaska	Basal stem decay	2015	KY200502	–	–	–	–
THF04	*B. cinerea*	Alaska	Foliar lesion	2014	KY200503	–	–	–	–

WBC01	*B. paeoniae*	Washington	Basal stem decay	2014	KY200504	–	–	–	–
WBC02	*B. paeoniae*	Washington	Basal stem decay	2014	KY200505	–	–	–	–
WBC03	*B. paeoniae*	Washington	Basal stem decay	2014	KY200506	–	–	–	–
WBC04	*B. paeoniae*	Washington	Basal stem decay	2014	KY200507	–	–	–	–
WBC04b	*B. cinerea*	Washington	Shoot dieback	2014	KY200508	–	–	–	–
WBC07	*B. cinerea*	Washington	Basal stem decay	2014	KY200509	–	–	–	–
WBC07b	WABot2	Washington	Leaf tip dieback	2014	KY200510	KY203903	KY211661	KY553208	KY230694
WBC09	*B. paeoniae*	Washington	Basal stem decay	2014	KY200511	–	–	–	–

[a](–) indicates gene was not sequenced or used in phylogenetic analysis

Supplementary Table S2 List of sequences used in phylogenetic analyses including GenBank accession numbers.

Isolate	Species	GenBank Accession Number[a]					Citation
		**G3PDH**	**HSP60**	**RPB2**	**NEP1**	**NEP2**	
MUCL8415	*B. aclada*	AJ704992	AJ716050	AJ745664	AM087059	AM087087	6, 24
PRI006		AJ704993	AJ716051	AJ745665	–	–	6
MUCL94	*B. byssoidea*	AJ704998	AJ716059	AJ745670	AM087045	AM087079	6, 24
X1487	*B. californica*	KJ937073	KJ937063	KJ937053	–	–	18
X503		KJ937068	KJ937058	KJ937048	–	–	18
X655		KJ937069	KJ937059	KJ937049	*	*	18
CBS175.63	*B. calthae*	AJ704999	AJ716060	AJ745671	–	–	6
MUCL1089		AJ705000	AJ716061	AJ745672	–	–	6
MUCL2830		AJ705001	AJ716062	AJ745673	AM087031	AM087088	6, 24
CA3	*B. caroliniana*	JF811586	JF811589	JF811592	–	–	15
CB15		JF811584	JF811587	JF811590	JF811593	†	15
WM4		JF811585	JF811588	JF811591	–	–	15
B05.10	*B. cinerea*	**	**	**	DQ211824	DQ211825	24
Bc21		AM231158	AM232675	AM231317	–	–	6
MUCL87		AJ705004	AJ716065	AJ745676	–	–	6
9801	*B. convoluta*	AJ705007	AJ716068	AJ745679	–	–	6
MUCL11595		AJ705008	AJ716069	AJ745680	AM087035	AM087062	6, 24
MUCL436	*B. croci*	AJ705009	AJ716070	AJ745681	AM087047	AM087065	6, 24
CBS 134649	*B. deweyae*	HG799521	HG799519	HG799518	HG799527	HG799520	6, 24
Be0006	*B elliptica*	AM231166	AM232664	AM231322	–	–	6
Be9605		AM231168	AM232668	AM231318	–	–	6
Be9714		AJ705012	AJ716073	AJ745684	AM087049	AM087080	6, 24
CERC 7163	*B. eucalypti*	KX301019	KX301023	KX301027	–	–	16
CERC 7170		KX301020	KX301024	KX301028	KX301032	KX301036	16
CERC 7208		KX301021	KX301025	KX301029	–	–	16
AK10	*B. euroamericana*	KX266727	KX266733	KX266739	–	–	13

B83		KC191677	KC191678	KC191679	–	–	13
HA06		KX266728	KX266734	KX266740	KX266746	KX266752	13
CBS109.57	*B. fabae*	AJ705013	AJ716074	AJ745685	–	–	6
MUCL98		AJ705014	AJ716075	AJ745686	DQ211829	DQ211831	6, 24
BroadbeanBC-13	*B. fabiopsis*	EU563109	EU563100	EU563115	–	–	21
BroadbeanBC-2		EU519211	EU514482	EU514473	†	†	21
BroadbeanBC-30		EU563106	EU563097	EU563117	–	–	21
CBS176.63	*B. ficariarum*	AJ705015	AJ716076	AJ745687	AM087055	AM087085	6, 24
MUCL376		AJ705016	AJ716077	AJ745688	–	–	6
D11_H_R4	*B. fragariae*	KX429702	KX429695	KX429709	–	–	17
U14_G2		KX429700	KX429693	KX429707	–	–	17
U14_P1		KX429699	KX429692	KX429706	KX429713	KX429720	17
MUCL3204	*B. galanthina*	AJ705017	AJ716078	AJ745690	AM087058	AM087067	6, 24
MUCL435		AJ705018	AJ716079	AJ745689	–	–	6
9701	*B. gladiolorum*	AJ705019	AJ716080	AJ745691	AJ716080	AJ745691	6, 24
MUCL3865		AJ705020	AJ716081	AJ745692	–	–	6
MUCL21514	*B. globosa*	AJ705021	AJ716082	AJ745694	AM087044	AM087070	6, 24
MUCL444		AJ705022	AJ716083	AJ745693	–	–	6
0001	*B. hyacinthi*	AJ705023	AJ716084	AJ745695	AM087048	AM087066	6, 24
MUCL442		AJ705024	AJ716085	AJ745696	–	–	6
BPI412756	*B. mali*	EF367129	–	–	–	–	44
MUCL18857	*B. narcissicola*	AJ705025	AJ716086	AJ745698	–	–	6
MUCL2120		AJ705026	AJ716087	AJ745697	AM087046	AM087078	6, 24
0003	*B. paeoniae*	AJ705027	AJ716088	AJ745699	AM087032	AM087064	6, 24
MUCL16084		AJ705028	AJ716089	AJ745700	–	–	6
CBS497.50	*B. pelargonii*	AJ704990	AJ716046	AM087030	–	–	6
MUCL1152		AJ705029	AJ716090	AJ745701	DQ211833	DQ211834	6, 24
CBS287.38	*B. polyblastis*	AJ705030	AJ716091	AJ745702	AM087039	AM087074	6, 24
MUCL3234	*B. porri*	AJ705032	AJ716093	AJ745704	AM087060	AM087063	6, 24
MUCL3349		AJ705033	AJ716094	AJ745705	–	–	6

Bpru-1.5	*B. prunorum*	KP339984	KP339998	KP339991	–	–	11
Bpru-1.9		KP339985	KP339999	KP339992	KR732663	KR425427	11
Bpru-49		KP339981	KP339995	KP339988	–	–	11
10091	*B. pseudocinerea*	JN692414	JN692400	JN692428	–	–	19
ICMP19668		KC620370	KC620323	–	–	–	33
ICMP19669		KC620375	KC620315	–	–	–	33
ICMP19670		KC620371	KC620318	–	–	–	33
ICMP19671		KC620372	KC620319	–	–	–	33
ICMP19672		KC620368	KC620320	–	–	–	33
ICMP19673		KC620367	KC62032	–	–	–	33
ICMP19674		KC620365	KC620325	–	–	–	33
ICMP19675		KC620366	KC620324	–	–	–	33
ICMP19676		KC620373	KC620317	–	–	–	33
ICMP19677		KC620374	KC620316	–	–	–	33
ICMP19681		KC620369	KC620322	–	–	–	33
VD256		JF421574	JX266722	–	–	–	19
SedsarBC-1	*B. pyriformis*	KJ543484	KJ543488	KJ543492	–	–	10
SedsarBC-2		KJ543485	KJ543489	KJ543493	–	–	10
SedsarBC-3		KJ543486	KJ543490	KJ543494	–	–	10
CBS178.63	*B. ranunculi*	AJ705034	AJ716095	AJ745706	AM087054	AM087086	6, 24
LeekBC-18	*B. sinoallii*	FJ169651	FJ169660	FJ169679	–	–	22
OnionBC-23		EU519217	EU514488	EU514479	–	–	22
OnionBC-59		FJ169646	FJ169658	FJ169678	–	–	22
GBC-3-1c	*B. sinoviticola*	JN692410	JN692396	JN692424	–	–	23
GBC-3-3c		JN692412	JN692398	JN692426	–	–	23
GBC-5		JN692413	JN692399	JN692427	†	†	23
MUCL21481	*B. sphaerosperma*	AJ705035	AJ716096	AJ745708	AM087042	AM087068	6, 24
MUCL21482		AJ705036	AJ716097	AJ745709	–	–	6
MUCL1107	*B. squamosa*	AJ705037	AJ716098	AJ745710	AM087052	AM087084	6, 24
MUCL9112		AJ705038	AJ716099	AJ745711	–	–	6

PRI026		AJ705039	AJ716100	AJ745707	–	–	6
Bt9701	*B. tulipae*	AM231172	AM232681	AM231325	–	–	6
Bt9806		AM231175	AM232682	AM231328	–	–	6
Bt9830		AJ705041	AJ716102	AJ745713	AM087037	AM087077	6, 24
bot079	*Botrytis spp.*	–	EU386596	–	–	–	31
bot080	*Botrytis spp.*	–	EU386597	–	–	–	31
bot093	*Botrytis spp.*	–	EU386598	–	–	–	31
bot095	*Botrytis spp.*	–	EU386599	–	–	–	31
bot109	*Botrytis spp.*	–	EU386600	–	–	–	31
bot1093	*Botrytis spp.*	–	EU386601	–	–	–	31
bot360	*Botrytis spp.*	–	EU386603	–	–	–	31
bot361	*Botrytis spp.*	–	EU386604	–	–	–	31
bot378	*Botrytis spp.*	–	EU386605	–	–	–	31
DAN5	*Botrytis spp.*	–	‡	–	–	–	–
DAN39	*Botrytis spp.*	–	‡	–	–	–	–
1980	*Sclerotinia sclerotiorum*	**	**	**	**	†	–

[a](–) indicates sequences that are either unavailable or not used in analyses. (*) indicates sequences that were generated for the purposes of this study from an isolate generously shared by Chang-Lin Xiao but was not submitted to GenBank. (**) indicates sequences was built from whole genome sequences publicly available in GenBank. (†) indicates sequences that were generously shared by Dr. Matthias Hahn. (‡) indicates sequences that were generously shared by Dr. Jan van Kan.
